# LPS activates neuroinflammatory pathways to induce depression in Parkinson’s disease-like condition

**DOI:** 10.3389/fphar.2022.961817

**Published:** 2022-10-06

**Authors:** Jing Zhang, Bing Xue, Bin Jing, Huiling Tian, Naiwen Zhang, Mengyuan Li, Lihua Lu, Lin Chen, Huaqiong Diao, Yufei Chen, Min Wang, Xiaoli Li

**Affiliations:** ^1^ Third Affiliated Hospital, Beijing University of Chinese Medicine, Beijing, China; ^2^ Core Facility Center, Capital Medical University, Beijing, China; ^3^ School of Biomedical Engineering, Capital Medical University, Beijing, China; ^4^ School of Traditional Chinese Medicine, Capital Medical University, Beijing, China

**Keywords:** Parkinson’s disease, depression, rat model, LPS, microglia, neuroinflammation, MRI

## Abstract

**Aim:** This study aimed to observe the effects of lipopolysaccharide (LPS) intraperitoneal (i.p.) injection on rats and investigate how neuroinflammation contributes to the pathogenesis of depression in Parkinson’s disease (dPD).

**Methods:** Rats were administered LPS (0.5 mg/kg, i.p.) for either 1, 2, or 4 consecutive days to establish a rat model of dPD. The sucrose preference test (SPT), the open field test (OFT), and the rotarod test evaluated depression-like and motor behaviors. Magnetic resonance imaging was used to detect alterations in the intrinsic activity and the integrity of white matter fibers in the brain. The expression of c-Fos, ionized calcium-binding adapter molecule (Iba-1), and tyrosine hydroxylase (TH) was evaluated using immunohistochemistry. The concentration of interleukin-6 (IL-6), tumor necrosis factor (TNF-α), and interleukin-10 (IL-10) was measured using Luminex technology.

**Results:** LPS i.p. injections decreased sucrose preference in the SPT, horizontal and center distance in the OFT, and standing time in the rotarod test. The intrinsic activities in the hippocampus (HIP) were significantly reduced in the LPS-4 d group. The integrity of white matter fibers was greatly destroyed within 4 days of LPS treatment. The expression of c-Fos and Iba-1 in the prefrontal cortex, HIP, and substantia nigra increased dramatically, and the number of TH^+^ neurons in the substantia nigra decreased considerably after LPS injection. The levels of IL-6, TNF-α, and IL-10 were higher in the LPS-4 d group than those in the control group.

**Conclusion:** Injection of LPS (0.5 mg/kg, i.p.) for 4 consecutive days can activate microglia, cause the release of inflammatory cytokines, reduce intrinsic activities in the HIP, destroy the integrity of white matter fibers, induce anhedonia and behavioral despair, and finally lead to dPD. This study proved that LPS injection (0.5 mg/kg, i.p.) for 4 consecutive days could be used to successfully create a rat model of dPD.

## 1 Introduction

Depression is a common complication of Parkinson’s disease (PD); approximately 30–40% of patients with PD have depression (Menon et al., 2015). Depressive symptoms are related to the severity of dyskinesia and may appear earlier than dyskinesia. Depression in PD (dPD) could damage the abilities of daily living and accelerate the progression of PD (Song et al., 2014). At present, the pathogenesis of dPD is not clear; however, substantial experimental evidence has shown that neuroinflammation can induce dPD (Felger et al., 2020; M. Guo et al., 2020). The activation of glial cells (especially microglia and astrocytes) causes the release of various inflammatory cytokines and neurotoxic factors (J. Guo et al., 2021). The neuronal loss will eventually lead to dPD. These findings encourage researchers to explore the pathogenesis of dPD from a broader perspective.

Establishing a successful animal model of dPD is crucial for its study and treatment. For the analysis of the dPD animal model, most researchers have used 6-hydroxydopamine, 1-methyl-4-phenyl-1,2,3,6-tetrahydropyridine, and rotenone to establish a PD model (Airavaara et al., 2020). A model of depression model was established mainly through chronic restraint stress or chronic unpredictable mild stress (CUMS) (Hao et al., 2019). However, these modeling methods are used primarily to simulate PD and depression symptoms but cannot fully explain the pathogenesis of dPD from the perspective of neuroinflammation. In contrast, injecting animals with lipopolysaccharide (LPS) is largely recognized and used to investigate the mechanism of inflammation. Much experimental evidence has confirmed that LPS activates glial cells (especially microglia) and causes the release of various inflammatory cytokines and neurotoxic factors, leading to neuroinflammation and neuronal loss in dPD (Kobayashi et al., 2013; Walker et al., 2014; M. Guo et al., 2020). In the presence of microglia, LPS-induced dopaminergic neuronal toxicity was twice as high as that induced by 6-hydroxydopamine, and tyrosine hydroxylase (TH)^+^ neurons were significantly more sensitive to LPS neurotoxicity than were TH^−^ neurons (O'Neill et al., 2020; Parra et al., 2020).

Because microglial activation plays a crucial role in neuroinflammation, we speculated that it might play a critical role in the pathogenesis of dPD. Therefore, this study adopted the LPS i.p. injection to create a model of dPD in rats and focused on evaluating microglial activation through the observation of ethology, neuroimaging through magnetic resonance imaging (MRI) technology, pathological morphological changes, and inflammatory cytokines. The dPD rat model was characterized in detail to provide a theoretical basis for the pathogenesis and treatment of dPD.

## 2 Materials and methods

### 2.1 Animals

Male Sprague-Dawley rats weighing 180–200 g (*n* = 42) were obtained from the Animal Experiment Centre of Capital Medical University, Beijing, China. Before starting the experiment, the rats were fed under standard feeding conditions (room temperature of 25°C, relative humidity 50%, 12-h light/dark cycle), and provided free access to food and water. The experiment was approved by the Experimental Animal Welfare and Ethics Committee of Capital Medical University (Animal Ethical Clearance Number: AEEI-2021-091).

### 2.2 Chemicals and instruments

LPS (*Escherichia coli*, serotype 055:B5) was purchased from Sigma-Aldrich (St. Louis, MO, United States). Thermo Pierce TM BCA Protein Assay Kit was purchased from Thermo Scientific (Kit Lot: TH269576; Waltham, MA, United States). MILLIPLEX^®^ Rat Cytokine/Chemokine Magnetic Bead Panel RECYTMAG-65K was purchased from Merck Millipore (Kit Lot: 3828876; Burlington, MA, United States). c-Fos antibody (ab208942) was purchased from Abcam (Cambridge, United Kingdom). Tyrosine hydroxylase (TH) antibody (25859-1-AP), ionized calcium-binding adapter molecule (Iba-1) antibody (10904-1-AP), secondary antibodies, and horseradish peroxidase-streptavidin (PK10006) were purchased from Proteintech (Rosemont, IL, United States).

### 2.3 Animal grouping and modeling

**FIGURE 1:**
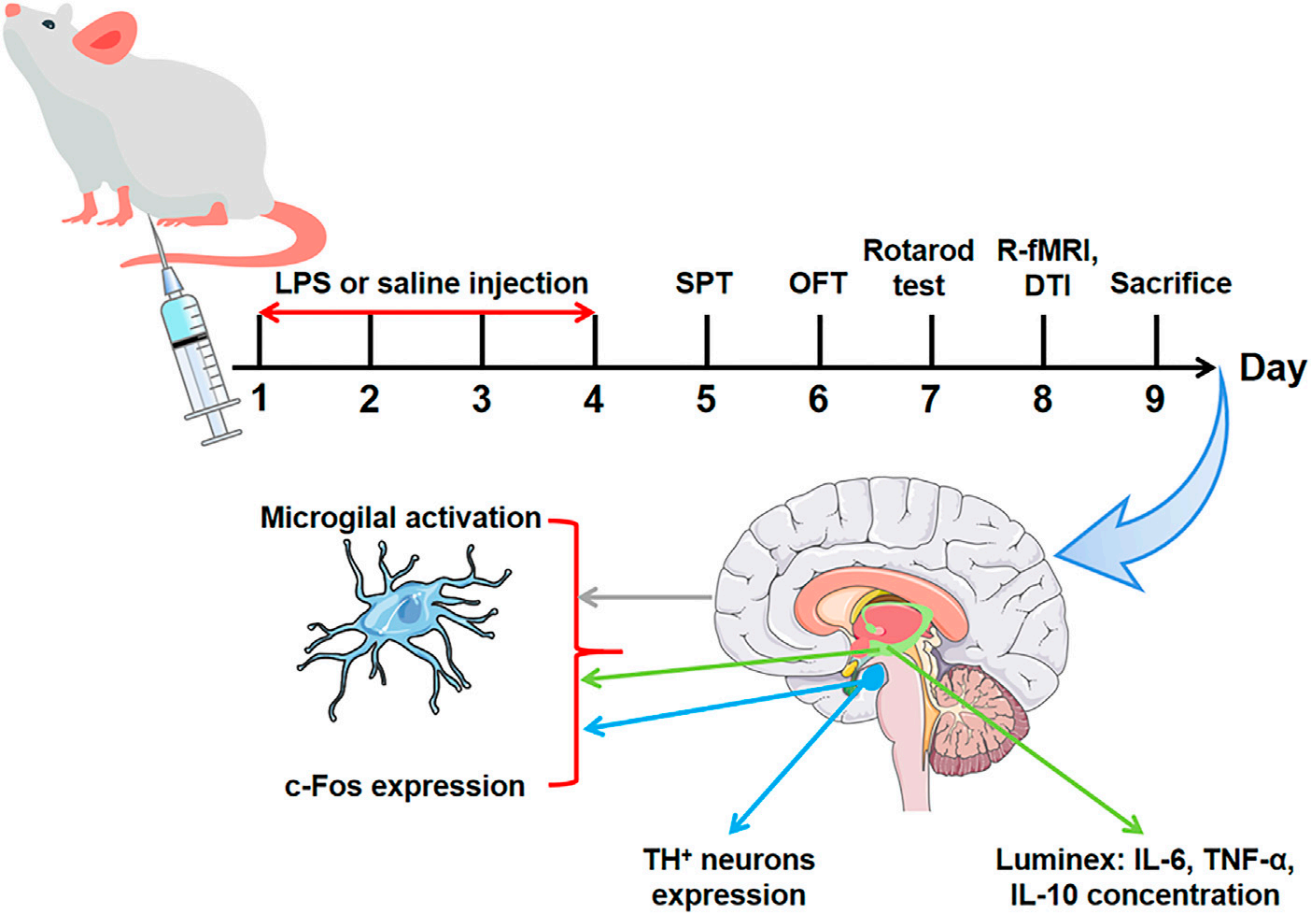
Experimental flow diagram.

Forty-two Sprague-Dawley rats were adaptively fed for 3 days, which was followed by an open field test (OFT). The rats whose autonomic activity times deviated significantly from the average were excluded. The remaining rats (*n* = 40) were randomly divided into four groups (*n* = 10/group): the control group (Control), LPS i.p. injection for 1 day group (LPS-1 d), LPS i.p. injection for 2 days group (LPS-2 d), and LPS i.p. injection for 4 days group (LPS-4 d). LPS was dissolved in sterile 0.9% saline at a 1 mg/ml concentration and injected into the rats at a dosage of 0.5 mg/kg, i.p., for 1 day or 2 or 4 consecutive days to create the dPD rat model. The dose was based on previously reported studies (Cui et al., 2018). In addition, rats in the control group were given a corresponding volume of 0.9% saline in the same manner. Twenty-four hours after the last LPS injection, all rats were subjected to behavioral and MRI testing, and samples were taken immediately after completion. The specific process is shown in Figure 1.

### 2.4 Behavioral tests

The four groups were subjected to the sucrose preference test (SPT), OFT, and the rotarod test 24 h after the last injection of LPS to verify the effect of LPS on depression-like and motor behaviors.

#### 2.4.1 Sucrose preference test

The SPT was used to test the animals’ reactivity to reward. For the first 24 h, all rats were given two bottles containing a 1% sucrose solution; for the second 24 h, the rats were provided with a bottle of water and a bottle containing 1% sucrose solution. At the 12th hour, the two bottles’ positions were changed. All rats were then deprived of water for 24 h, and the rats were given a bottle of 1% sucrose solution and a bottle of pure water. After 12 h, the bottles were removed, weighed, and the consumption of sucrose solution and pure water consumption was recorded.

#### 2.4.2 Open field test

The OFT was used to evaluate rats’ exploratory behavior and spontaneous movement ability. The experiment was conducted in a quiet environment and the rats were allowed to acclimatize to the laboratory for at least 10 min. The rats were placed in the center of the bottom in the open field reaction box and the cameras and timing equipment were set up. All movement distances of each rat within 5 min and movement distances to the central area were observed and recorded. After the experiment, each rat was wiped clean with alcohol to avoid the influence of residual odor on the next rat. After the OFT of all rats, the test results were recorded and statistical processing of the experimental data were accomplished with SuperMaze 2.0 (Xinruan Information and Technology, Shanghai, China).

#### 2.4.3 Rotarod test

The rotarod test requires animals to keep balance on a rotating axis and move continuously. The test is a widely used experiment to test the coordination of movement. The Rat RotaRod NG (47750; Ugo Basile, Gemonio, Italy) is composed of a rotating shaft and four independent compartments. Each rat was trained four times, 3 days before the experiment. In the formal rod rotation experiment, rats were placed on the rod rotation apparatus for 10 s and the recording was started when their gait was stable and balance was maintained. The rotation speed was accelerated from 5 rpm/min to 40 rpm/min within 1 min, and the standing time was recorded. If the rats did not fall, the maximum testing period was 300 s. After each detection, the rats were put back into the cage to rest for 1 min and tested seven times consecutively. The maximum and minimum values were removed and the values of the remaining five times were averaged.

### 2.5 Magnetic resonance imaging

#### 2.5.1 Magnetic resonance imaging data acquisition

Twelve rats (six in the control group and six in the LPS-4 d group), were included in the current study. All animals were anesthetized using an isoflurane vaporizer set at 5% for induction and 2% during the MRI scanning. Data were acquired using a 7.0 T MR scanner (Bruker BioSpec GmbH, Rheinstetten, Germany) at the Capital Medical University. The following sequences were acquired: T2-weighted rapid acquisition with relaxation enhancement [Repetition time (TR): 5600 ms, echo time (TE): 36 ms, rare factor (RF): 8, slice thickness: 1 mm, slice gap: 0, number of averages: 2, field of view (FOV): 3.2 cm × 3.2 cm, matrix size: 256 × 256 voxels], the resting-state functional magnetic resonance imaging (R-fMRI) (TR: 2000 ms, TE: 13 ms, flip angle: 90°, slice thickness: 1 mm, slice gap: 0, number of averages: 1, FOV: 2.5 cm × 2.0 cm, matrix size: 80 × 64 voxels) and the echo-planar diffusion tensor imaging (DTI) (TR: 6250 ms, TE: 23 ms, b-value: 1000 s/mm^2^, 30 diffusion directions, four segments, 1/δ: 5 ms, slice thickness: 1 mm, interslice distance: 0 mm, number of averages: 1, FOV: 3.3 cm × 3.3 cm, matrix size: 128 × 128 voxels). Each rat’s structural MRI scanning procedure lasted about 10 min, then the R-fMRI scanning (13 min and 50 s) and the DTI scanning (16 min and 40 s) were started. Each rat’s respiration was continuously measured with a pressure transducer placed on the abdomen below the ribcage during the scanning. The isoflurane might be slightly adjusted to maintain a safe respiration rate (40–50 breaths/min).

#### 2.5.2 Resting-state functional magnetic resonance imaging data processing

Data processing was performed using the Data Processing & Analysis for Brain Imaging (DPABI) (Yan et al., 2016) and Statistical Parametric Mapping (SPM12, https://www.fil.ion.ucl.ac.uk/spm) tools. The voxel dimensions of anatomical and echo-planar images were scaled by a constant factor of 10 to optimize their utilization in standard neuroimaging software packages designed for human imaging. Then, the first 10 volumes of echo-planar images were discarded to allow magnetization to reach a steady state. After slice-timing correction, all volumes were realigned to the first volume, and all rats displayed maximal translational and rotational head motion less than 2 mm or 2°. The echo-planar images were then co-registered into the Paxinos template. After that, the images were smoothed with a full width-half maximum kernel at 2 mm and filtered by a 0.01–0.1 Hz band-pass filter. Finally, regression of six head motion parameters and linear trends were conducted to minimize the influence of nuisance covariates.

#### 2.5.3 Amplitude of low-frequency fluctuations analysis

We computed the amplitude of low-frequency fluctuations (ALFF) for each rat. First, the time series was converted to the frequency domain using a fast Fourier transform algorithm, and the averaged square root across 0.01–0.08 Hz (in contrast with the whole frequency domain) was used to compute ALFF at each voxel (Zang et al., 2007). Finally, ALFF was transformed to a Z score to improve the normality for subsequent group comparison.

#### 2.5.4 Statistical analysis

T-tests for two independent samples were performed to determine the ALFF differences between the control and LPS-4 d groups. The statistical threshold was set at the individual voxel *p* < 0.005 with a cluster size > 6.

#### 2.5.5 Diffusion tensor imaging analysis

DTI was processed with ParaVision Version 5.1 (RRID: SCR_001964) software. Subsequently, the fractional anisotropy (FA), mean diffusivity (MD), and the tracer of nerve fibers were created. Regions of interest were drawn on each slice to segment the bilateral hippocampus (HIP), bilateral prefrontal cortex (PFC), bilateral ventral tegmental area (VTA), and dorsal raphe nucleus (DRN). The average FA and MD values were calculated for the segmented bilateral HIP, bilateral PFC, bilateral VTA, and DRN.

### 2.6 Immunohistochemistry

Four-micrometer coronal brain sections were sliced using a cryostat (Thermo Fisher, United States). The brain sections were immunostained using standard avidin-biotin immunohistochemical protocols. The paraffin slices were sequentially soaked in the following: xylene I and xylene II for 15 min to remove the paraffin and 100% ethanol I, 100% ethanol II, 95% ethanol, and 80% ethanol for 5 min each. The sections were rinsed with phosphate-buffered saline three times for 3 min and then rinsed with distilled water for 1 min. The slices were incubated with antigen retrieval reagent, peroxidase suppressor, and 5% goat serum for 30 to block nonspecific binding. Then the sections were incubated for one night with a specific primary antibody: a rat monoclonal c-Fos antibody (Abcam, United Kingdom) diluted 1:1000, a rat polyclonal anti-TH (Proteintech, United States) diluted 1:500, and a rat polyclonal anti-allograft inflammatory factor 1 (Iba-1) (Proteintech) diluted 1:400. After careful washing (3 × phosphate-buffered saline rinses), the sections were incubated first with the secondary antibody (Proteintech) at room temperature for 1 h, HRP-streptavidin (Proteintech), and diaminobenzidine (DAB) (0.5 mg/ml) complex for 5 min. After being washed, the sections were mounted on gelatin-coated slides, air-dried, dehydrated in ascending ethanol concentrations, cleared with xylene, and coverslipped under neutral gum. Controls underwent the same procedures confirm the specificity of the primary and secondary antibodies. The slices were observed using a microscope, and the average optical density (AOD) was analyzed with ImageJ software (v. 1.8.0; RRID: SCR_003070; NIH, United States). Immunohistochemical analysis was performed by two experimenters blinded to reduce any perceived bias.

### 2.7 Luminex

The HIP was homogenized and the homogenate was used to determine protein concentration with the BCA kit. The concentrations of IL-6, TNF-α, and IL-10 in the HIP were determined with a Luminex instrument according to the manufacturer’s instructions. The specific steps were as follows. Briefly, the steps were as follows. The Luminex instrument uses 96-well plates. A standard curve was generated using 25 μl of standards or controls (assay buffer for the 0 ng/ml standard) in the appropriate wells. Diluted (1:2) HIP samples (25 μl) were added to the appropriate wells. Mixed beads were added to each well. The plate was sealed, wrapped with foil, and incubated with agitation for 2 h at 20–25°C. Streptavidin-phycoerythrin was added to each well containing the 25 μl of detection antibodies. The plate was sealed, covered, and agitated as in the earlier step, except for only 30 min. The well contents were removed. The plate was washed and the Sheath Fluid PLUS (125 μl) was added to all wells. The beads were resuspended on a plate shaker for 5 min. The plate was then run on a Luminex instrument. The mean fluorescent intensity data was analyzed using a 5-parameter logistic or spline curve-fitting method to calculate the IL-6, TNF-α, and IL-10 concentrations in the samples.

### 2.8 Statistical analysis

GraphPad Prism 9 (v.9; RRID:SCR_002798; San Diego, CA, United States) was used for statistical analysis. Differences between groups were tested using one-way analysis of variance followed by the Tukey’s post hoc test or Brown-Forsythe analysis of variance test followed by Dunnett’s T3 multiple comparisons test if the data were not normally distributed. The data are expressed as the mean ± SEM. *p* < 0.05 was considered statistically significant.

## 3 Results

### 3.1 Behavioral tests results

**FIGURE 2:**
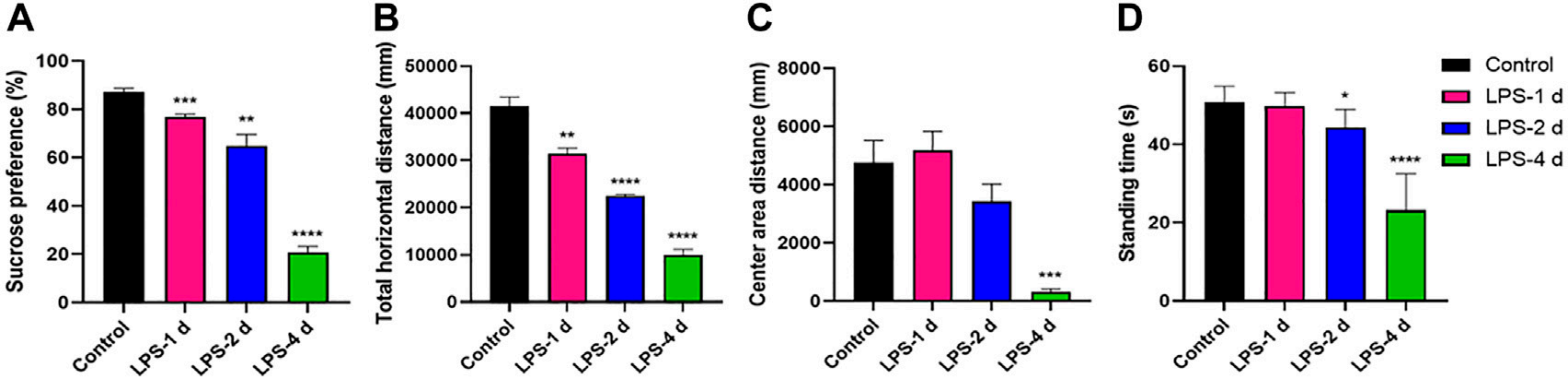
Behavioral tests results. **(A)** Sucrose preference in the sucrose preference test (SPT). **(B)** Total horizontal distance (mm) in the open field test (OFT). **(C)** Center area distance (mm) in OFT. **(D)** Standing time (s) in the rotarod test. Data are expressed as the mean ± SEM (n = 10).*p < 0.05, **p < 0.01, ***p < 0.005, ****p < 0.001, compared to the control group.

Significant differences in sucrose preference (%) across groups were observed in the SPT (F = 110.8, *p* < 0.0001, Figure 2A). The total horizontal distance in the OFT (F = 105.4, *p* < 0.0001, Figure 2B), center area distance in the OFT (F = 14.1, *p* < 0.0001, Figure 2C), and the standing time in the rotarod test (F = 48.89, *p* < 0.0001, Figure 2D) were significantly different. Multiple comparisons suggested that the sucrose preference (%) (*p* < 0.0001, Figure 2A), the total horizontal distance (*p* < 0.0001, Figure 2B) and center area distance (*p* = 0.0008, Figure 2C), and the standing time (*p* < 0.0001, Figure 2D) were lower in the LPS-4 d group than those in the control group. The sucrose preference (%) (*p* = 0.0023, Figure 2A), the total horizontal distance (*p* < 0.0001, Figure 2B), and the standing time (*p* = 0.0109, Figure 2D) were significantly lower in the LPS-2 d group than those in the control group, whereas there was not statistically significant difference in the center area distance. The sucrose preference (%) (*p* = 0.0001, Figure 2A) and the total horizontal distance (*p* = 0.0018, Figure 2B) were also lower in the LPS-1 d group than those in the control group; other results were not statistically significant.

### 3.2 Intrinsic activity alterations

**FIGURE 3:**
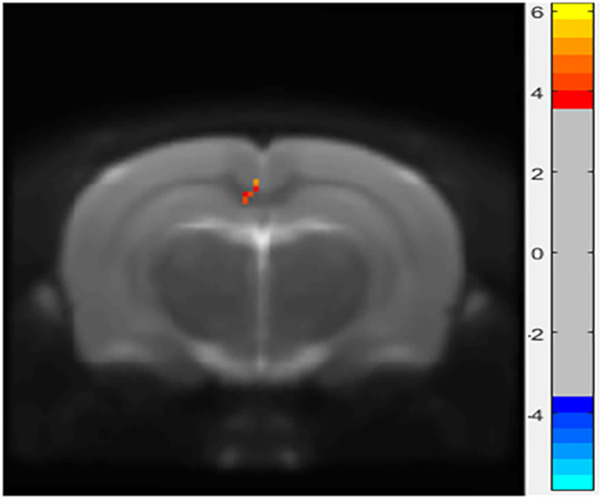
Differences in amplitude of low-frequency oscillations (ALFF) between the control and LPS-4 d groups. The statistical threshold was set at cluster size > 6, which corresponds to a corrected *p* < 0.005.

In the ALFF analysis of the HIP, intrinsic activity alterations indexed by ALFF were shown to be significantly lower in the LPS-4 d group than those in the control group (*p* < 0.005, cluster size > 6, Figure 3).

### 3.3 Diffusion tensor imaging results

**FIGURE 4:**
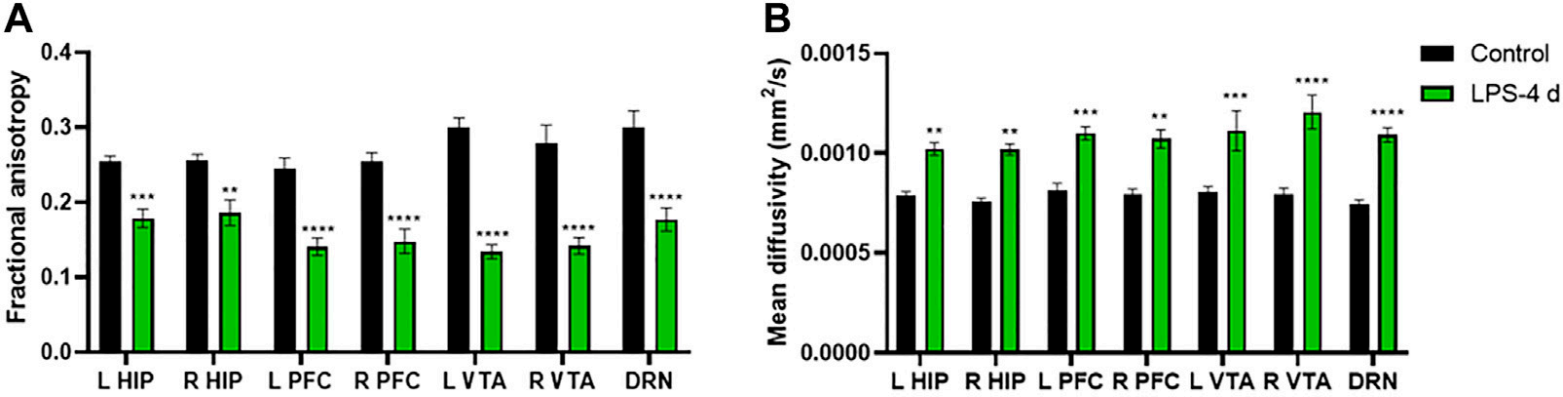
Diffusion tensor imaging (DTI) analysis of the bilateral hippocampus (L HIP, R HIP), prefrontal cortex (L PFC, R PFC), ventral tegmental area (L VTA, R VTA), and dorsal raphe nucleus (DRN) in the control group and the LPS-4 d group. **(A)** Fractional anisotropy (FA) in the control group and the LPS-4 d group. **(B)** Mean diffusivity (MD) in the control group and the LPS-4 d group. Data are expressed as the mean ± SEM (*n* = 6). **p < 0.01, ***p < 0.005, ****p < 0.001, compared to the control group.

**FIGURE 5:**
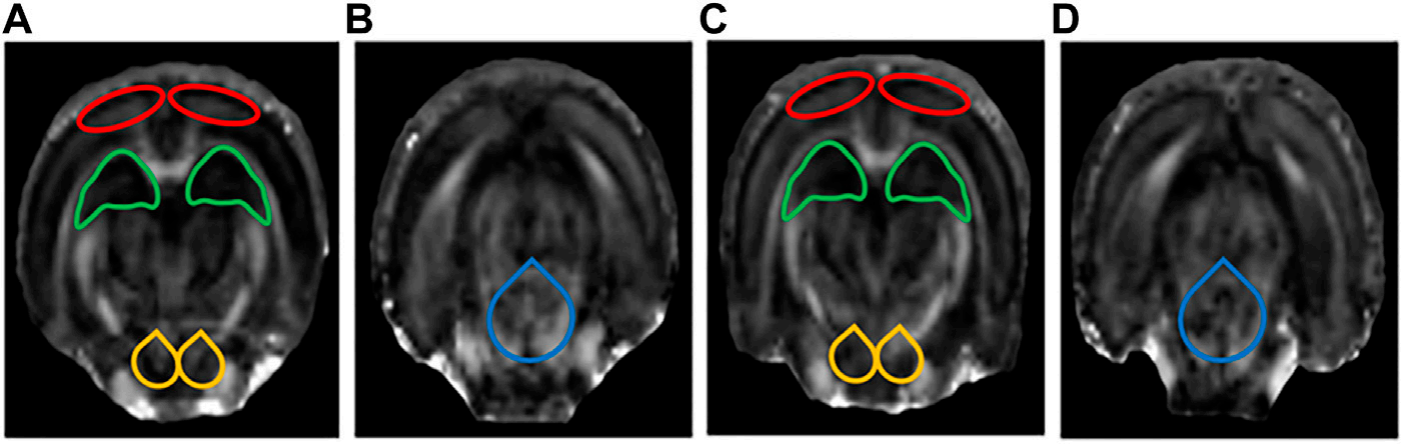
Display diagram of FA values (*n* = 6). **(A)** Display diagram of FA values in the prefrontal cortex (PFC), hippocampus (HIP), and ventral tegmental area (VTA) in the control group. **(B)** Display diagram of FA values in the dorsal raphe nucleus (DRN) in the control group. **(C)** Display diagram of FA values in the PFC, HIP, and VTA in the LPS-4 d group. **(D)** Display diagram of FA values in the DRN in the LPS-4 d group. The red outlines indicated the PFC, the green outlines indicated the HIP, the yellow outlines indicated the VTA, and the blue outlines indicated the DRN.

**FIGURE 6:**
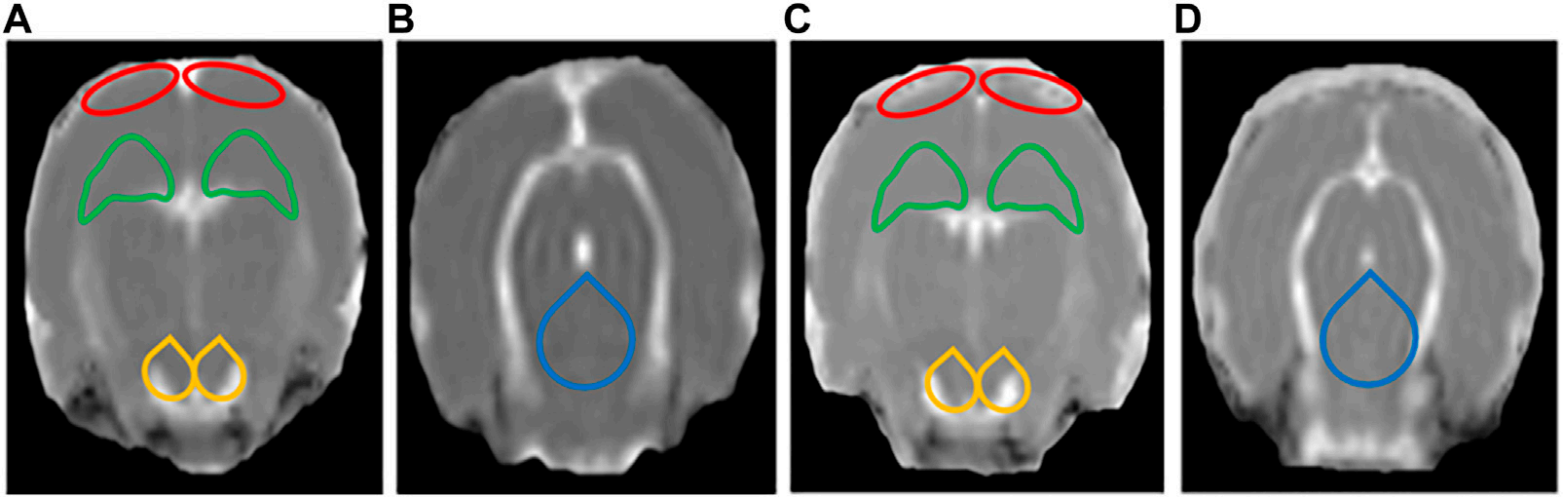
Display diagram of MD values (*n* = 6). **(A)** Display diagram of MD values in the prefrontal cortex (PFC), hippocampus (HIP), and ventral tegmental area (VTA) in the control group. **(B)** Display diagram of MD values in the dorsal raphe nucleus (DRN) in the control group. **(C)** Display diagram of MD values in the PFC, HIP, and VTA in the LPS-4 d group. **(D)** Display diagram of MD values in the DRN in the LPS-4 d group. The red outlines indicated the PFC, the green outlines indicated the HIP, the yellow outlines indicated the VTA, and the blue outlines indicated the DRN.

**FIGURE 7:**
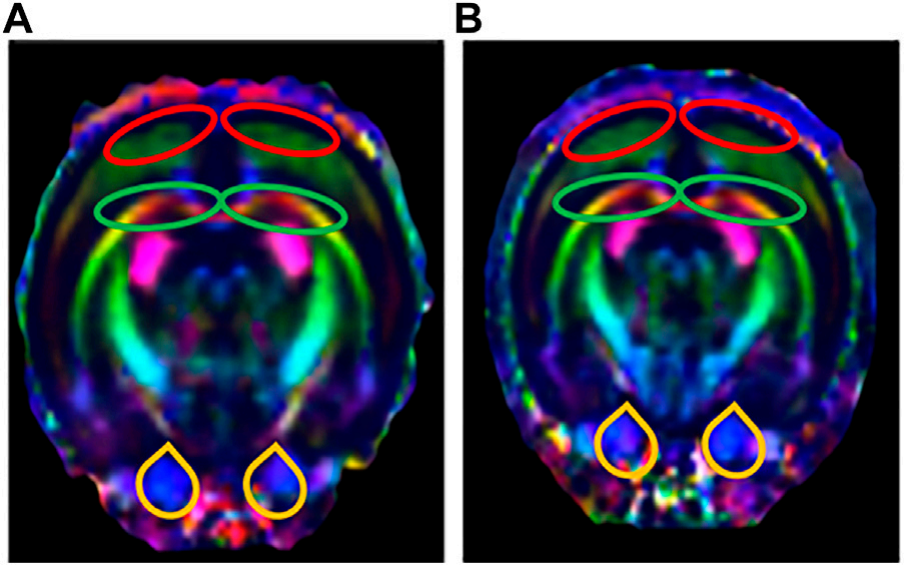
Tracing of nerve fibers (*n* = 6). The direction of travel: blue, rostral to caudal; Green, dorsal and ventral; Red, left and right. **(A)** Tracing of nerve fibers in the control group. **(B)** Tracing of nerve fibers in the LPS-4 d group. The red outlines indicated the prefrontal cortex (PFC), the green outlines indicated the hippocampus (HIP), and the yellow outlines indicated the ventral tegmental area (VTA).

The DTI results showed that the FA values of the bilateral HIP, the bilateral PFC, the bilateral VTA, and the DRN of rats in the LPS-4 d group were significantly lower than those in the control group (F = 285, *p* < 0.0001; Left HIP, *p* = 0.0009; Right HIP, *p* = 0.002; *p* < 0.0001 for Left PFC, Right PFC, Left VTA, Right VTA, and DRN; Figure 4A). In contrast, the MD values were significantly higher (F = 147.2, *p* < 0.0001; Left HIP, *p* = 0.0077; Right HIP, *p* = 0.0026; Left PFC, *p* = 0.0009; Right PFC, *p* = 0.0014; Left VTA, *p* = 0.0003; Right VTA and DRN, *p* < 0.0001; Figure 4B). Figures 5, 6 show the display diagram of FA values and MD values in the control and LPS-4 d groups. Figure 7 shows the tracing of nerve fibers in the control and LPS-4 d groups.

### 3.4 c-Fos expression

**FIGURE 8:**
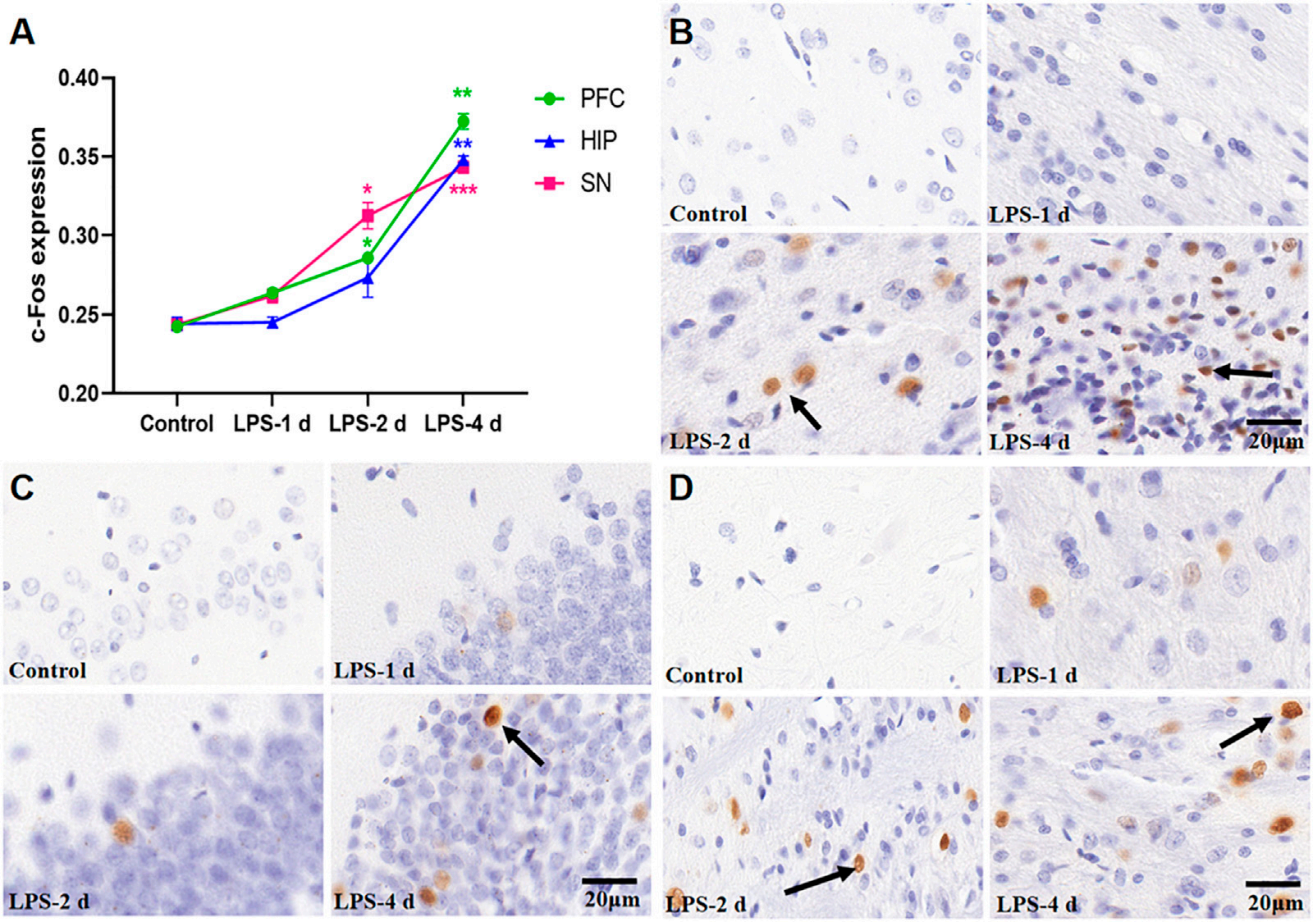
c-Fos expression in the PFC, HIP, and SN for all groups (*n* = 3). **(A)** The average optical density (AOD) value of each group was compared. Data are expressed as the mean ± SEM. *p < 0.05, **p < 0.01, ***p < 0.005, compared to the control group. **(B)** Representative photomicrographs of c-Fos expression in the PFC (×400). **(C)** Representative photomicrographs of c-Fos expression in the HIP (×400). **(D)** Representative photomicrographs of c-Fos expression in the SN (×400). The arrows showed the expression of c-Fos.

Figure 8 depict the expression of c-Fos in the PFC, HIP, and substantia nigra (SN) for all groups. These groups had distinctly significant differences (F = 278.7, *p* < 0.0001, Figure 8). There was no statistical significance in the c-Fos expression in the PFC, HIP, and SN in the LPS-1 d group compared to the control group. The c-Fos expression in the PFC (*p* = 0.0153) and SN (*p* = 0.0316) was significantly higher in the LPS-2 d group than that in the control group; there was no statistical significance in the HIP. Moreover, the c-Fos expression in the PFC (*p* = 0.0013), HIP (*p* = 0.0069), and SN (*p* = 0.0001) were significantly higher in the LPS-4 d group than that in the control group.

### 3.5 Ionized calcium-binding adapter molecule 1 expression

**FIGURE 9:**
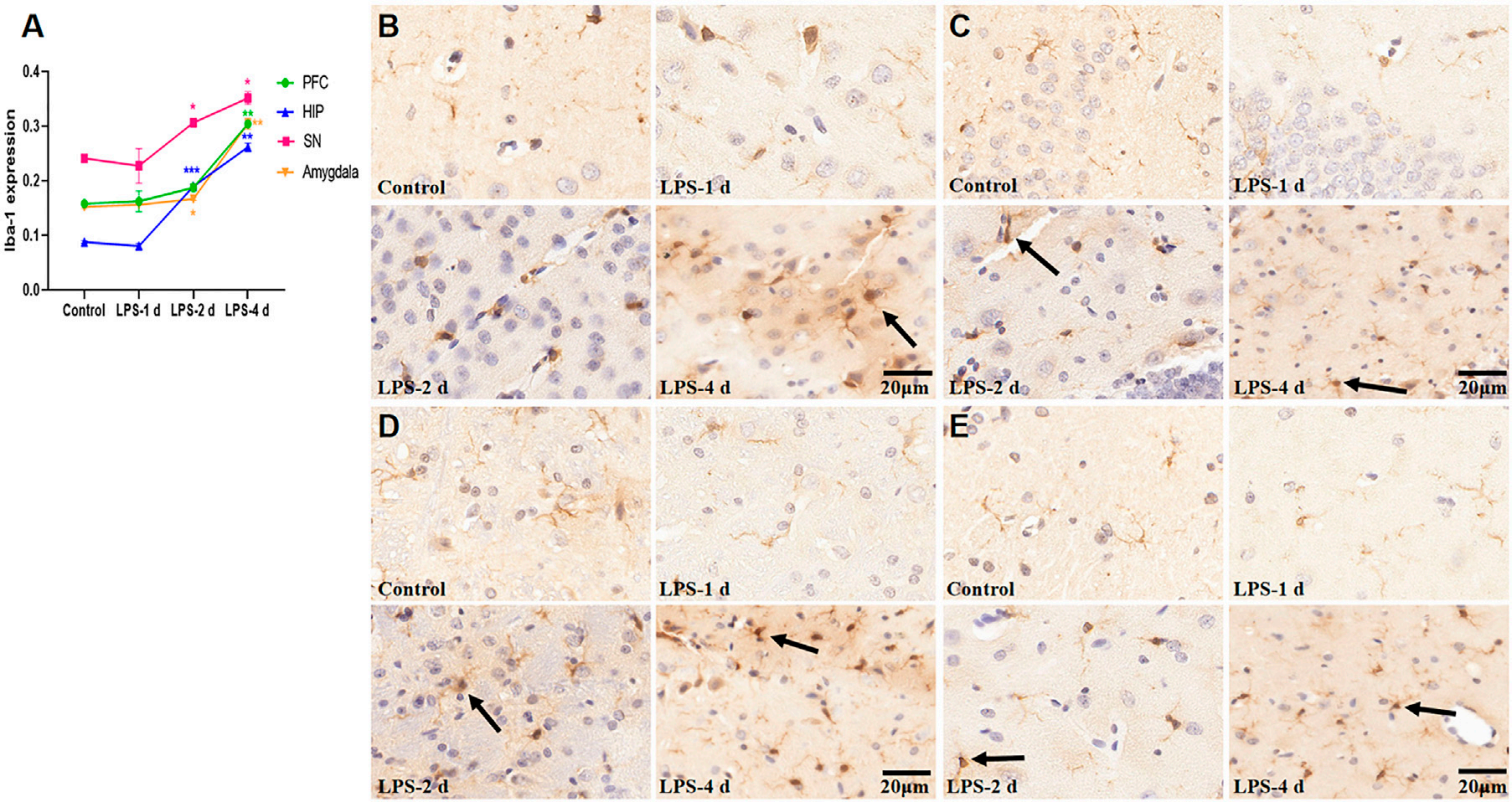
Ionized calcium-binding adapter molecule (Iba-1) expression in the PFC, HIP, SN, and amygdala for all groups (*n* = 3). **(A)** The AOD value of each group was compared. Data are expressed as the mean ± SEM. *p < 0.05, **p < 0.01, ***p < 0.005, compared to the control group. **(B)** Representative photomicrographs of Iba-1 expression in the PFC (×400). **(C)** Representative photomicrographs of Iba-1 expression in the HIP (×400). **(D)** Representative photomicrographs of Iba-1 expression in the SN (×400). **(E)** Representative photomicrographs of Iba-1 expression in the amygdala (×400). The arrows showed the expression of activated microglia.

Significant differences in Iba-1 expression were observed across groups (F = 192.2, *p* < 0.0001, Figure 9). There was no statistical significance in Iba-1 expression in the PFC, HIP, SN, and amygdala in the LPS-1 d group compared to that in the control group. Iba-1 expression in the HIP (*p* = 0.0003), SN (*p* = 0.0142), and amygdala (*p* = 0.0315) was significantly higher in the LPS-2 d group than that in the control group, whereas there was no statistical significance in the PFC. In the LPS-4 d group, Iba-1 expression in the PFC (*p* = 0.0084), HIP (*p* = 0.002), SN (*p* = 0.0311), and amygdala (*p* = 0.0099) was dramatically higher than that in the control group.

### 3.6 TH^+^ neurons

**FIGURE 10:**
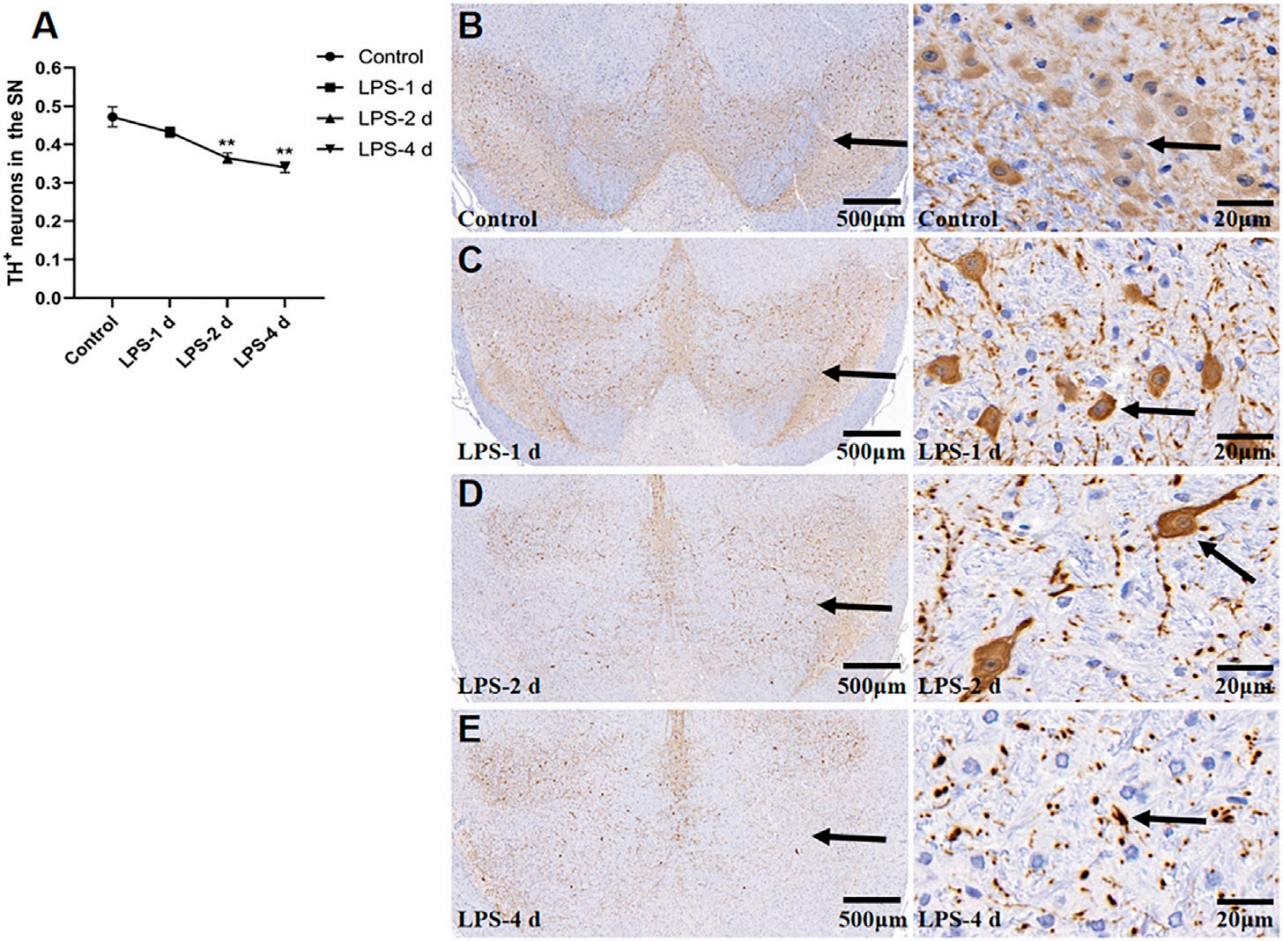
Tyrosine hydroxylase (TH)^+^ neurons in the SN for all groups (*n* = 3). **(A)** The AOD value of each group was compared. Data are expressed as the mean ± SEM. **p < 0.01, compared to the control group. **(B)** Representative photomicrographs of TH^+^ neurons in the control group (Left: ×16, Right: ×400). **(C)** Representative photomicrographs of TH^+^ neurons in the LPS-1 d group (Left: ×16, Right: ×400). **(D)** Representative photomicrographs of TH^+^ neurons in the LPS-2 d group (Left: ×16, Right: ×400). **(E)** Representative photomicrographs of TH^+^ neurons in the LPS-4 d group (Left: ×16, Right: ×400). The arrows showed the expression of TH^+^ neurons.

**FIGURE 11:**
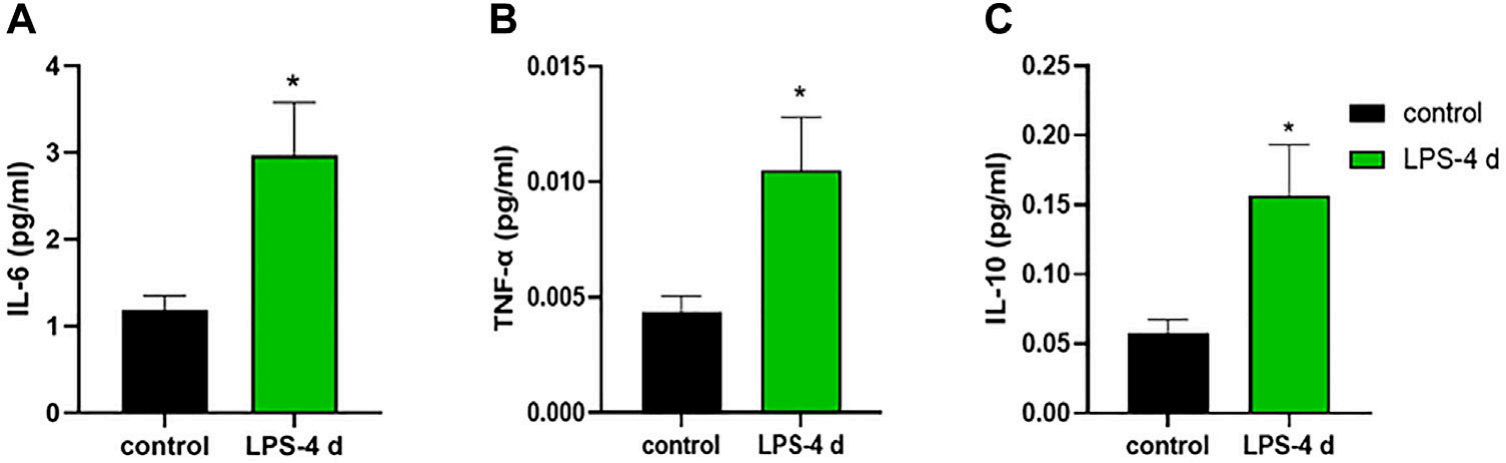
Levels of interleukin (IL)-6, tumor necrosis factor (TNF)-α, and interleukin (IL)-10. **(A)** IL-6 level in the control group and the LPS-4 d group. **(B)** TNF-α level in the control group and the LPS-4 d group. **(C)** IL-10 level in the control group and the LPS-4 d group. Data are expressed as the mean ± SEM (*n* = 6). *p < 0.05, compared to the control group.

Figure 10 depict the TH^+^ neuron staining and TH^+^ expression in the SN for all groups (F = 12.25, *p* = 0.0025, Figure 11). In the SN, LPS injection (i.p.) caused a 23% (*p* = 0.0062) reduction of TH^+^ neurons at 2 d and 28% (*p* = 0.0018) at 4 d compared to the control group. However, the LPS-1 d group showed no statistical significance in the number of TH^+^ neurons when compared to the control group.

### 3.7 Concentrations of interleukin-6, tumor necrosis factor, and interleukin-10

The unpaired t-test indicated that in the HIP, the LPS-4 d group had higher concentrations of IL-6 (t = 2.842, *p* = 0.0175, Figure 11A), TNF-α (t = 2.561, *p* = 0.0283, Figure 11B), and IL-10 (t = 2.642, *p* = 0.0247, Figure 11C) than that in the control group.

## 4 Discussion

Depression is common and often associated with PD (Kano et al., 2011). Depression is not only a risk factor for PD but is also closely related to the progression and severity of PD (Gustafsson et al., 2015). Depressive symptoms occur in about 40% of patients with PD (Jacob et al., 2010). Furthermore, about 20% of patients are already depressed when they are diagnosed with PD (Shiba et al., 2000). Animal studies can help to understand the pathogenesis of dPD so that more effective treatments can be sought. Many scholars believe that systemic inflammation causes chronic nervous system inflammation. Immune abnormalities and inflammatory responses in the central nervous system are involved in impairing dopaminergic neurons in SN, which leads to progressive neurodegenerative diseases (Felger et al., 2020). Substantial experimental evidence suggests that neuroinflammation can induce PD and depression (M. Guo et al., 2020), and that the level of inflammatory cytokines is positively correlated with the severity of depression (Husain et al., 2017). The LPS animal model of dPD may be a better representation of neuroinflammation. LPS has not been used to induce dPD directly in animal models for analysis. Scholars often use LPS to induce PD, and then use CUMS to induce depression, which does not well reflect the pathogenesis of dPD, since depressive symptoms may appear earlier than motor disturbances. On the other hand, central injections of LPS are technically demanding, and it is uncertain whether the damage to neurons is caused by LPS alone or in combination with mechanical damage. Therefore, this study adopted the LPS i.p. injection to create a model of dPD in rats through the observation of ethology, MRI technology, pathological morphological changes, and inflammatory cytokines, which better determined the time point of PD and depression in rats, simulated the pathogenesis of dPD and provided a theoretical basis for the pathogenesis of dPD.

Many animal models of LPS-induced PD have been studied, and LPS intraventricular injection can induce a PD animal model successfully. In one study, LPS injection into the striatum caused an approximately 48% reduction in TH^+^ neurons at 7 days post-infection, and the expression of Iba-1 in the striatum increased by 86%, whereas they increased by 36% in the 6-hydroxydopamine-treated group (Parra et al., 2020). In a rat model with PD, an injection of LPS into the right SN pars compacta led to microglial activation and TH^+^ neurons lost (Huang et al., 2017). Early work reported that i.p. injection of LPS (2 mg/kg) into rats did not disrupt blood-brain barrier permeability, suggesting that i.p. injection of LPS is unlikely to affect the central nervous system (M. Liu and Bing, 2011). However, other studies have found that PD animal models can also be prepared by i.p. injection of LPS. A previous study (O'Neill et al., 2020) reported that systemic LPS injection (0.25 mg/kg, i.p.) exacerbated the deficits in skilled motor functions in rats previously lesioned with intranigral LPS and led to a 22% decrease in TH^+^ neurons within the SN. Moreover, the systemic LPS injection aggravated microglial activation and IL-1β expression. In another study (S. Y. Wu et al., 2011), the expression of Iba-1 increased as early as 3 h after LPS (1 mg/kg) i.p. injection and lasted for longer than 3 weeks. Additionally, the decrease of TH^+^ neurons was significant on day 1 and lasted for 5 weeks after the LPS injection. Meanwhile, LPS i.p. injection reduces striatal dopamine content levels and impaired motor coordination. Some studies (Park et al., 2015; Kang et al., 2022) have confirmed that the expression of Iba-1 in the SN increased by more than twofold following systemic injection of LPS (10 mg/kg). The expression of inflammatory factors in the SN was also significantly increased in mice treated with LPS as a single injection (5 mg/kg) (Yao et al., 2015). Some studies have shown that a single systemic inflammatory response will transfer to the brain and induce persistent neuroinflammation lasting 7 months, leading to the gradual loss of dopaminergic neurons in the SN (Qin et al., 2007; Zheng et al., 2013). In addition, intraperitoneal LPS injection (0.25, 0.5, or 1 mg/kg) is commonly used to induce animal models of depression. It was reported that LPS reduced the sucrose preference in the SPT and the number of crossings in the OFT, and it increased immobility duration in a forced swimming experiment (Cui et al., 2018; Carabelli et al., 2020; Zhang et al., 2020; Zhao et al., 2020).

In this study, we chose i.p. injections of LPS as the modeling technique. Our results showed that the sucrose preference in SPT, the total horizontal distance and center area distance in OFT, and the standing time in the rotarod test in the LPS-4 d group were significantly lower than those in the control group. Daily i.p. injections of LPS for 4 days led to microglial activation and a significant loss (28%) of TH^+^ neurons in the SN. Additionally, the dopaminergic neurons were more damaged in the LPS-4 d group (28%) than in the LPS-2 d group (23%), an effect that gradually increased over time. These results were in agreement with previous findings (Zheng et al., 2013; Parra et al., 2020). Moreover, the DTI results showed that the FA values of the bilateral VTA in the LPS-4 d group were significantly lower than those in the control group, whereas the MD values were significantly higher. This finding demonstrated that LPS induced damage to white matter fibers in the VTA, which is one of our innovations. The above results showed that LPS (0.5 mg/kg, i.p.) injected for 4 days successfully established the dPD rat model. The innovation is that our modeling method is easier to perform, takes less time, induces behavioral, histopathological, and white matter integrity changes, and simulates the pathogenetic process of dPD in this rat model of dPD.

R-fMRI is easy to perform and does not require many subjects; therefore, it can also be applied to rodents to provide standard data across species (Y. Liu et al., 2020). ALFF is one of the main evaluation indexes of R-fMRI; it can directly maintain the amplitude of fMRI signal changes relative to the baseline and reflect alterations in intrinsic activity and energy in local brain regions. Previous fMRI studies have shown that patients with dPD mainly have gray matter atrophy in the prefrontal-limbic system (Valli et al., 2019) and abnormal functional networks in the PFC (Zhu et al., 2016) and HIP (Sheng et al., 2014). Animal studies (Y. Luo et al., 2014; Williams et al., 2014; Q. Li et al., 2022) have shown that rats with depression-like behavior showed significant abnormalities in the striatum and HIP. The regional homogeneity of bilateral caudate putamen and nucleus accumbens regions was increased in LPS-injected rats (Ji et al., 2019).

In this study, the R-fMRI results showed that the ALFF in the HIP of the LPS-4 d group was significantly lower than that in the control group. This is consistent with previous research (Y. Luo et al., 2014; Williams et al., 2014; Q. Li et al., 2022) and may be related to the release of inflammatory cytokines causing neuronal damage, thus affecting neuronal activity. c-Fos is an intranuclear phosphorylated protein resulting from the transcription and translation of the *c-Fos* gene. Because c-Fos protein expression can broadly reflect stimulus-induced neuronal activity, it is often used as a marker of neuronal activation (Alfonso-Gonzalez and Riesgo-Escovar, 2018). In this study, c-Fos immunohistochemistry showed that an i.p. injection of LPS can activate neurons in the PFC, HIP, and SN. This result may indicate that an i.p. injection of LPS can activate neurons briefly by releasing inflammatory cytokines. Stimulated by prolonged inflammation, neurons are damaged, and, subsequently, the intrinsic activities decline. This finding could also explain the R-fMRI results.

DTI is the only non-invasive method to detect the connectivity and integrity of white matter fiber tracts at the molecular level. The commonly used indicators are FA and MD; the FA value can provide images with high white matter contrast, and the MD value indicates the average rate of diffusion of water molecules in tissues and reflects the degree of diffusion. Studies have shown that the integrity of deep brain white matter fibers is destroyed in patients with dPD (J. Y. Wu et al., 2018), and the PFC (C. Luo et al., 2016) and HIP (van Mierlo et al., 2015) are atrophied to varying degrees. The DTI results showed that the FA values of the bilateral HIP, the bilateral PFC, the bilateral VTA, and the DRN in the LPS-4 d group were significantly lower than those in the control group, whereas the MD values were significantly higher. These findings demonstrated that LPS not only induced damage to white matter fibers in the HIP and PFC, which was consistent with previous research (van Mierlo et al., 2015; C. Luo et al., 2016), but it also induced damage to white matter fibers in the VTA and DRN.

When the brain is stimulated, glial cells in the brain will produce inflammatory responses, which is a common characteristic of dPD pathology. Neuroinflammatory responses characterized by microglial activation are an essential step in this process (Husain et al., 2017). LPS is the most widely used activator of microglia-induced inflammatory responses and is the natural ligand of toll-like receptor 4 (TLR-4), which is widely expressed in microglia. LPS injected peripherally does not cross the blood-brain barrier; however, it does directly activate cultured brain microglia by binding TLR4, which induces a robust microgliosis and a high degree of microglial activation (Angelopoulou et al., 2020). Studies (Kobayashi et al., 2013; Walker et al., 2014) have shown that LPS can directly induce M1-phenotype microglia and release inflammatory factors, such as TNF-α and IL-6 whereas IL-4 induces M2-phenotype microglia. After being activated by LPS, microglia release many inflammatory cytokines, which also activate astrocytes and cause the further release of inflammatory cytokines (Kwon and Koh, 2020). Inflammatory cytokines simultaneously act on both dopaminergic neurons and serotonergic neurons, leading to neuronal degeneration. Decreased neuronal activity leads to decreased or lost interest in—and reduced behavior motivated by—a rewarding stimulus, and this cyclic process ultimately exacerbates the occurrence and development of dPD (M. Liu and Bing, 2011; T. B. Li and Le, 2020). Moreover, the SN contains 4.5 times more microglia than other brain regions; it is therefore the region most susceptible to neuroinflammation. The loss of TH^+^ neurons in the SN is more evident than in other regions (Qin et al., 2007). Our study also showed that i.p. injection of LPS for 4 days led to microglial activation in PFC, HIP, SN, and amygdala. LPS injection also led to a significant loss of TH^+^ neurons in the SN.

Some studies (Kim et al., 2016; Mahajan et al., 2018) have shown that HIP neuroinflammation is involved in the pathogenesis of depression. Other studies (Qin et al., 2007; Engler et al., 2017; Wang and Miller, 2018; Zhang et al., 2020) suggest that IL-6 and TNF-α can lead to the damage and loss of neurons in the brain—effects that are related to dPD. Therefore, the concentrations of IL-6, TNF-α, and IL-10 in the HIP were measured in this study. After 4 days of daily i.p. injections of LPS, the levels of IL-6, TNF-α, and IL-10 were significantly higher than those in the control group. The increase in inflammatory cytokines was closely related to subsequent activation of microglia and destruction of TH^+^ neurons. These results may indicate that neuroinflammation caused by microglial activation is one of the critical steps involved in the pathogenesis of dPD. Reducing inflammation by inhibiting the activation of microglia should be the top priority in treating dPD in the future. However, our experiments also have some limitations. First, our study did not carry out follow-up observations over a long period and did not look at whether other organs were affected by LPS-induced systemic inflammation, requiring a more expansive and rigorous design in the future. Second, the levels of dopamine and serotonin in cerebrospinal fluid were not measured. In addition, specific M1 and M2 markers of microglia would have proven the predominance of inflammatory phenotype, which required further studies in the future.

## 5 Conclusion

The current investigation indicates that LPS injection (0.5 mg/kg, i.p.) for 4 consecutive days could be used to successfully create a model of dPD in rats. The underlying mechanism may be that LPS activates microglia, leading to the release of inflammatory cytokines, chronic and persistent neuroinflammation, damage to dopaminergic neurons in the brain, and, ultimately, dPD. These results suggest that inhibiting microglial activation and reducing the inflammatory response may be one of the targets for the development of therapeutics for dPD.

## Data Availability

The original contributions presented in the study are included in the article/Supplementary Materials; further inquiries can be directed to the corresponding author.

## Supplementary material

Supplementary Figure S1 A voxel based morphometry study between the control and LPS-4 d groups. Two sample t-test with FDR correction, P < 0.05, cluster size > 20. Refer to SIGMA rat brain template. The red area represents the brain area with a larger volume in the control group than in the LPS-4 d group. The blue area represents the brain area that was smaller in the control group than in the LPS-4 d group.

## References

[B1] Airavaara M. Parkkinen I. Konovalova J. Albert K. Chmielarz P. Domanskyi A. (2020). Back and to the future: From neurotoxin-induced to human Parkinson's disease models. Curr. Protoc. Neurosci. 91 (1), e88. 10.1002/cpns.88 32049438

[B2] Alfonso-Gonzalez C. Riesgo-Escovar J. R. (2018). Fos metamorphoses: Lessons from mutants in model organisms. Mech. Dev. 154, 73–81. 10.1016/j.mod.2018.05.006 29753813

[B3] Angelopoulou E. Paudel Y. N. Shaikh M. F. Piperi C. (2020). Fractalkine (CX3CL1) signaling and neuroinflammation in Parkinson's disease: Potential clinical and therapeutic implications. Pharmacol. Res. 158, 104930. 10.1016/j.phrs.2020.104930 32445958

[B4] Carabelli B. Delattre A. M. Waltrick A. P. F. Araujo G. Suchecki D. Machado R. B. (2020). Fish-oil supplementation decreases Indoleamine-2, 3-Dioxygenase expression and increases hippocampal serotonin levels in the LPS depression model. Behav. Brain Res. 390, 112675. 10.1016/j.bbr.2020.112675 32407816

[B5] Cui Y. Yang Y. Ni Z. Dong Y. Cai G. Foncelle A. (2018). Astroglial Kir4.1 in the lateral habenula drives neuronal bursts in depression. Nature 554 (7692), 323–327. 10.1038/nature25752 29446379

[B6] Engler H. Brendt P. Wischermann J. Wegner A. Rohling R. Schoemberg T. (2017). Selective increase of cerebrospinal fluid IL-6 during experimental systemic inflammation in humans: Association with depressive symptoms. Mol. Psychiatry 22 (10), 1448–1454. 10.1038/mp.2016.264 28138158

[B7] Felger J. C. Haroon E. Patel T. A. Goldsmith D. R. Wommack E. C. Woolwine B. J. (2020). What does plasma CRP tell us about peripheral and central inflammation in depression? Mol. Psychiatry 25 (6), 1301–1311. 10.1038/s41380-018-0096-3 29895893 PMC6291384

[B8] Guo J. Zhang X. L. Bao Z. R. Yang X. K. Li L. S. Zi Y. (2021). Gastrodin regulates the notch signaling pathway and Sirt3 in activated microglia in cerebral hypoxic-ischemia neonatal rats and in activated BV-2 microglia. Neuromolecular Med. 23 (3), 348–362. 10.1007/s12017-020-08627-x 33095377

[B9] Guo M. Wang J. Zhao Y. Feng Y. Han S. Dong Q. (2020). Microglial exosomes facilitate alpha-synuclein transmission in Parkinson's disease. Brain 143 (5), 1476–1497. 10.1093/brain/awaa090 32355963 PMC7241957

[B10] Gustafsson H. Nordstrom A. Nordstrom P. (2015). Depression and subsequent risk of Parkinson disease: A nationwide cohort study. Neurology 84 (24), 2422–2429. 10.1212/WNL.0000000000001684 25995056 PMC4478031

[B11] Hao Y. Ge H. Sun M. Gao Y. (2019). Selecting an appropriate animal model of depression. Int. J. Mol. Sci. 20 (19), E4827. 10.3390/ijms20194827 31569393 PMC6801385

[B12] Huang B. Liu J. Ju C. Yang D. Chen G. Xu S. (2017). Licochalcone A prevents the loss of dopaminergic neurons by inhibiting microglial activation in lipopolysaccharide (LPS)-Induced Parkinson's disease models. Int. J. Mol. Sci. 18 (10), E2043. 10.3390/ijms18102043 28937602 PMC5666725

[B13] Husain M. I. Strawbridge R. Stokes P. R. Young A. H. (2017). Anti-inflammatory treatments for mood disorders: Systematic review and meta-analysis. J. Psychopharmacol. 31 (9), 1137–1148. 10.1177/0269881117725711 28858537

[B14] Jacob E. L. Gatto N. M. Thompson A. Bordelon Y. Ritz B. (2010). Occurrence of depression and anxiety prior to Parkinson's disease. Park. Relat. Disord. 16 (9), 576–581. 10.1016/j.parkreldis.2010.06.014 PMC2963655 20674460

[B15] Ji M. Mao M. Li S. Zhang L. Qiu L. Li B. (2019). Acute ketamine administration attenuates lipopolysaccharide-induced depressive-like behavior by reversing abnormal regional homogeneity in the nucleus accumbens. Neuroreport 30 (6), 421–427. 10.1097/WNR.0000000000001219 30855557

[B16] Kang S. Piao Y. Kang Y. C. Lim S. Pak Y. K. (2022). DA-9805 protects dopaminergic neurons from endoplasmic reticulum stress and inflammation. Biomed. Pharmacother. 145, 112389. 10.1016/j.biopha.2021.112389 34775235

[B17] Kano O. Ikeda K. Cridebring D. Takazawa T. Yoshii Y. Iwasaki Y. (2011). Neurobiology of depression and anxiety in Parkinson's disease. Park. Dis. 2011, 143547. 10.4061/2011/143547 PMC3109308 21687804

[B18] Kim Y. K. Na K. S. Myint A. M. Leonard B. E. (2016). The role of pro-inflammatory cytokines in neuroinflammation, neurogenesis and the neuroendocrine system in major depression. Prog. Neuropsychopharmacol. Biol. Psychiatry 64, 277–284. 10.1016/j.pnpbp.2015.06.008 26111720

[B19] Kobayashi K. Imagama S. Ohgomori T. Hirano K. Uchimura K. Sakamoto K. (2013). Minocycline selectively inhibits M1 polarization of microglia. Cell Death Dis. 4, e525. 10.1038/cddis.2013.54 23470532 PMC3613832

[B20] Kwon H. S. Koh S. H. (2020). Neuroinflammation in neurodegenerative disorders: The roles of microglia and astrocytes. Transl. Neurodegener. 9 (1), 42. 10.1186/s40035-020-00221-2 33239064 PMC7689983

[B21] Li Q. Zhao W. Liu S. Zhao Y. Pan W. Wang X. (2022). Partial resistance to citalopram in a Wistar-Kyoto rat model of depression: An evaluation using resting-state functional MRI and graph analysis. J. Psychiatr. Res. 151, 242–251. 10.1016/j.jpsychires.2022.04.010 35500452

[B22] Li T. B. Le W. D. (2020). Biomarkers for Parkinson's disease: How good are they? Neurosci. Bull. 36 (2), 183–194. 10.1007/s12264-019-00433-1 31646434 PMC6977795

[B23] Liu M. Bing G. (2011). Lipopolysaccharide animal models for Parkinson's disease. Park. Dis. 2011, 327089. 10.4061/2011/327089 PMC3096023 21603177

[B24] Liu Y. Perez P. D. Ma Z. Ma Z. Dopfel D. Cramer S. (2020). An open database of resting-state fMRI in awake rats. Neuroimage 220, 117094. 10.1016/j.neuroimage.2020.117094 32610063 PMC7605641

[B25] Luo C. Song W. Chen Q. Yang J. Gong Q. Shang H. F. (2016). Cortical thinning in drug-naive Parkinson's disease patients with depression. J. Neurol. 263 (10), 2114–2119. 10.1007/s00415-016-8241-x 27485171

[B26] Luo Y. Cao Z. Wang D. Wu L. Li Y. Sun W. (2014). Dynamic study of the hippocampal volume by structural MRI in a rat model of depression. Neurol. Sci. 35 (11), 1777–1783. 10.1007/s10072-014-1837-y 24929958

[B27] Mahajan G. J. Vallender E. J. Garrett M. R. Challagundla L. Overholser J. C. Jurjus G. (2018). Altered neuro-inflammatory gene expression in hippocampus in major depressive disorder. Prog. Neuropsychopharmacol. Biol. Psychiatry 82, 177–186. 10.1016/j.pnpbp.2017.11.017 29175309 PMC5801125

[B28] Menon B. Nayar R. Kumar S. Cherkil S. Venkatachalam A. Surendran K. (2015). Parkinson's disease, depression, and quality-of-life. Indian J. Psychol. Med. 37 (2), 144–148. 10.4103/0253-7176.155611 25969597 PMC4418244

[B29] O'Neill E. Yssel J. D. McNamara C. Harkin A. (2020). Pharmacological targeting of β2 -adrenoceptors is neuroprotective in the LPS inflammatory rat model of Parkinson's disease. Br. J. Pharmacol. 177 (2), 282–297. 10.1111/bph.14862 31506926 PMC6989960

[B30] Park W. Kang S. Piao Y. Pak C. Oh M. Kim J. (2015). Ethanol extract of Bupleurum falcatum and saikosaponins inhibit neuroinflammation via inhibition of NF-κB. J. Ethnopharmacol. 174, 37–44. 10.1016/j.jep.2015.07.039 26231448

[B31] Parra I. Martinez I. Ramirez-Garcia G. Tizabi Y. Mendieta L. (2020). Differential effects of LPS and 6-OHDA on microglia's morphology in rats: Implications for inflammatory model of Parkinson's disease. Neurotox. Res. 37 (1), 1–11. 10.1007/s12640-019-00104-z 31478124

[B32] Qin L. Wu X. Block M. L. Liu Y. Breese G. R. Hong J. S. (2007). Systemic LPS causes chronic neuroinflammation and progressive neurodegeneration. Glia 55 (5), 453–462. 10.1002/glia.20467 17203472 PMC2871685

[B33] Sheng K. Fang W. D. Su M. L. Li R. Zou D. Z. Han Y. (2014). Altered spontaneous brain activity in patients with Parkinson's disease accompanied by depressive symptoms, as revealed by regional homogeneity and functional connectivity in the prefrontal-limbic system. PLoS One 9 (1), e84705. 10.1371/journal.pone.0084705 24404185 PMC3880326

[B34] Shiba M. Bower J. H. Maraganore D. M. McDonnell S. K. Peterson B. J. Ahlskog J. E. (2000). Anxiety disorders and depressive disorders preceding Parkinson's disease: A case-control study. Mov. Disord. 15 (4), 669–677. 10.1002/1531-8257(200007)15:4<669::aid-mds1011>3.0.co;2-5 10928577

[B35] Song W. Guo X. Chen K. Chen X. Cao B. Wei Q. (2014). The impact of non-motor symptoms on the Health-Related Quality of Life of Parkinson's disease patients from Southwest China. Park. Relat. Disord. 20 (2), 149–152. 10.1016/j.parkreldis.2013.10.005 24161377

[B36] Valli M. Mihaescu A. Strafella A. P. (2019). Imaging behavioural complications of Parkinson's disease. Brain Imaging Behav. 13 (2), 323–332. 10.1007/s11682-017-9764-1 28856542

[B37] van Mierlo T. J. Chung C. Foncke E. M. Berendse H. W. van den Heuvel O. A. (2015). Depressive symptoms in Parkinson's disease are related to decreased hippocampus and amygdala volume. Mov. Disord. 30 (2), 245–252. 10.1002/mds.26112 25600157

[B38] Walker F. R. Beynon S. B. Jones K. A. Zhao Z. Kongsui R. Cairns M. (2014). Dynamic structural remodelling of microglia in health and disease: A review of the models, the signals and the mechanisms. Brain Behav. Immun. 37, 1–14. 10.1016/j.bbi.2013.12.010 24412599

[B39] Wang A. K. Miller B. J. (2018). Meta-analysis of cerebrospinal fluid cytokine and tryptophan catabolite alterations in psychiatric patients: Comparisons between schizophrenia, bipolar disorder, and depression. Schizophr. Bull. 44 (1), 75–83. 10.1093/schbul/sbx035 28338954 PMC5768046

[B40] Williams K. A. Mehta N. S. Redei E. E. Wang L. Procissi D. (2014). Aberrant resting-state functional connectivity in a genetic rat model of depression. Psychiatry Res. 222 (1-2), 111–113. 10.1016/j.pscychresns.2014.02.001 24613017

[B41] Wu J. Y. Zhang Y. Wu W. B. Hu G. Xu Y. (2018). Impaired long contact white matter fibers integrity is related to depression in Parkinson's disease. CNS Neurosci. Ther. 24 (2), 108–114. 10.1111/cns.12778 29125694 PMC6489851

[B42] Wu S. Y. Wang T. F. Yu L. Jen C. J. Chuang J. I. Wu F. S. (2011). Running exercise protects the substantia nigra dopaminergic neurons against inflammation-induced degeneration via the activation of BDNF signaling pathway. Brain Behav. Immun. 25 (1), 135–146. 10.1016/j.bbi.2010.09.006 20851176

[B43] Yan C. G. Wang X. D. Zuo X. N. Zang Y. F. (2016). Dpabi: Data processing & analysis for (Resting-State) brain imaging. Neuroinformatics 14 (3), 339–351. 10.1007/s12021-016-9299-4 27075850

[B44] Yao N. Wu Y. H. Zhou Y. Ju L. L. Liu Y. J. Ju R. K. (2015). Lesion of the locus coeruleus aggravates dopaminergic neuron degeneration by modulating microglial function in mouse models of Parkinson's disease. Brain Res. 1625, 255–274. 10.1016/j.brainres.2015.08.032 26342895

[B45] Zang Y. F. He Y. Zhu C. Z. Cao Q. J. Sui M. Q. Liang M. (2007). Altered baseline brain activity in children with ADHD revealed by resting-state functional MRI. Brain Dev. 29 (2), 83–91. 10.1016/j.braindev.2006.07.002 16919409

[B46] Zhang K. Liu R. Gao Y. Ma W. Shen W. D. (2020). Electroacupuncture relieves LPS-induced depression-like behaviour in rats through Ido-mediated tryptophan-degrading pathway. Neuropsychiatr. Dis. Treat. 16, 2257–2266. 10.2147/Ndt.S274778 33116524 PMC7547135

[B47] Zhao J. H. Liu X. J. Chang D. Y. Zhang X. T. Lian H. M. Du X. M. (2020). Low-dose ketamine improves LPS-induced depression-like behavior in rats by activating cholinergic anti-inflammatory pathways. ACS Chem. Neurosci. 11 (5), 752–762. 10.1021/acschemneuro.9b00669 32011849

[B48] Zheng H. F. Yang Y. P. Hu L. F. Wang M. X. Wang F. Cao L. D. (2013). Autophagic impairment contributes to systemic inflammation-induced dopaminergic neuron loss in the midbrain. PLoS One 8 (8), e70472. 10.1371/journal.pone.0070472 23936437 PMC3735600

[B49] Zhu Y. Song X. Xu M. Hu X. Li E. Liu J. (2016). Impaired interhemispheric synchrony in Parkinson's disease with depression. Sci. Rep. 6, 27477. 10.1038/srep27477 27265427 PMC4893739

