# Supplementary material for: LPS activates neuroinflammatory pathways to induce depression in Parkinson’s disease-like condition
Source: Front Pharmacol. 2022 Oct 6;13:961817. doi: 10.3389/fphar.2022.961817 (PMC9582846; doi:10.3389/fphar.2022.961817)

**Trajectory Chart**

**Control 1**


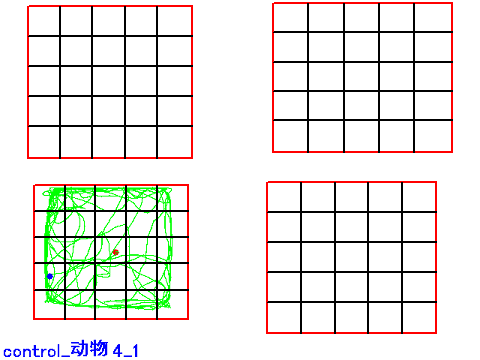


**Control 2**


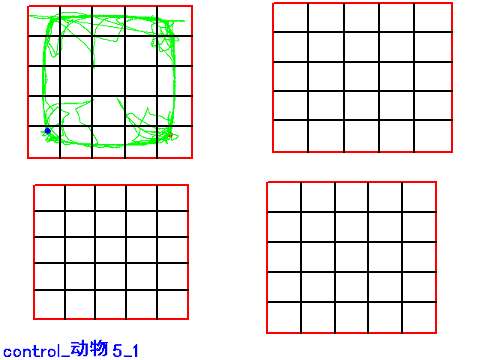


**Control 3**


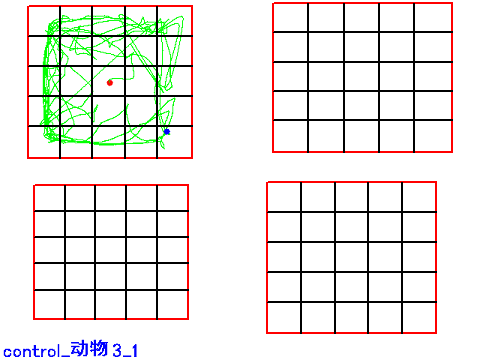


**Control 4**


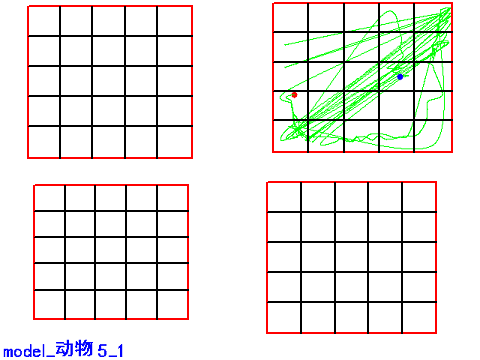


**Control 5**


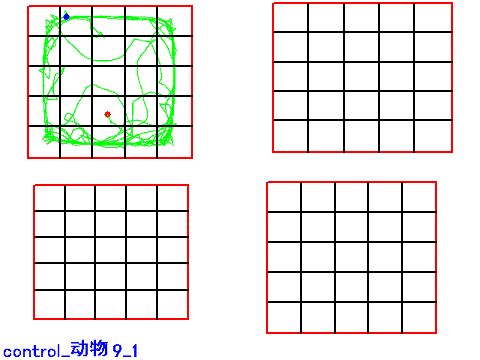


**Control 6**


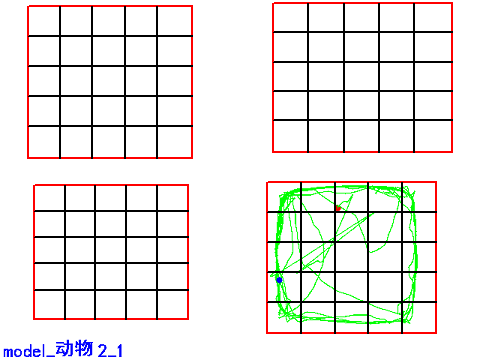


**Control 7**


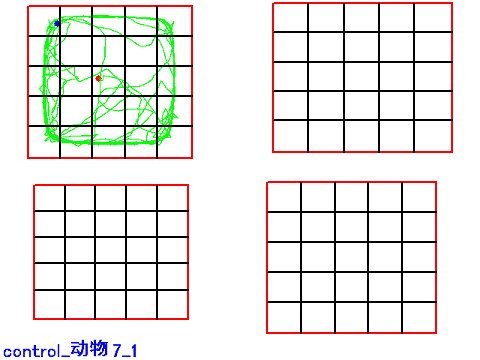


**Control 8**


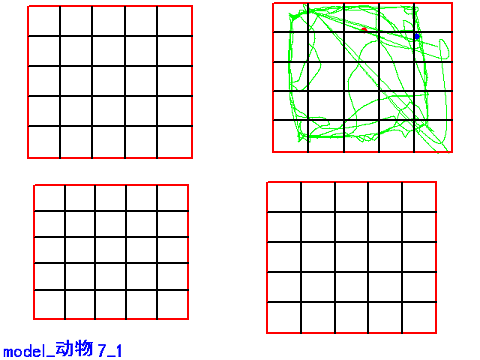


**Control 9**


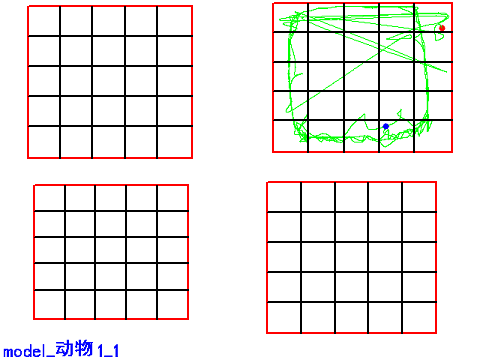


**Control 10**


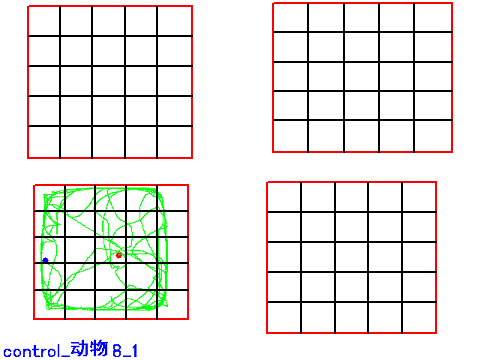


**LPS-1 d 1**


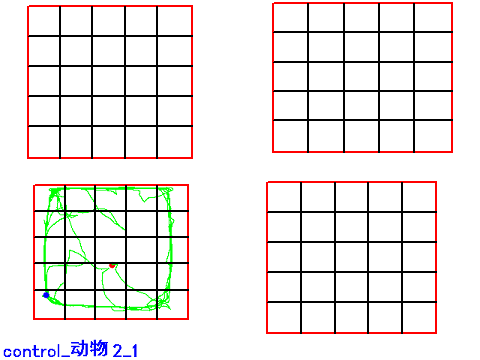


**LPS-1 d 2**


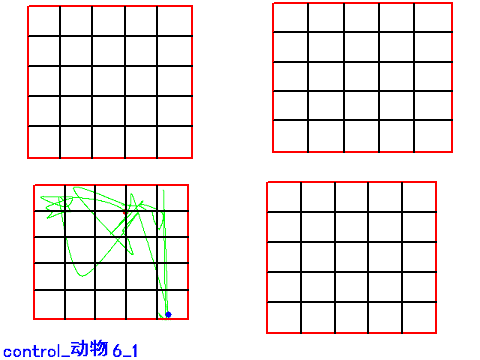


**LPS-1 d 3**


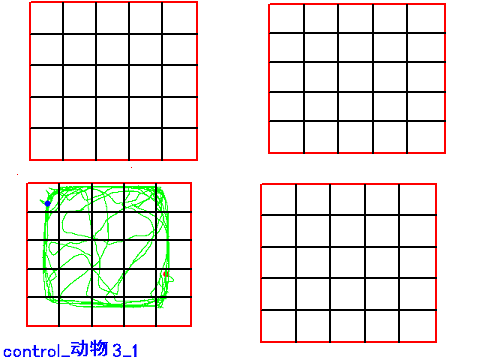


**LPS-1 d 4**


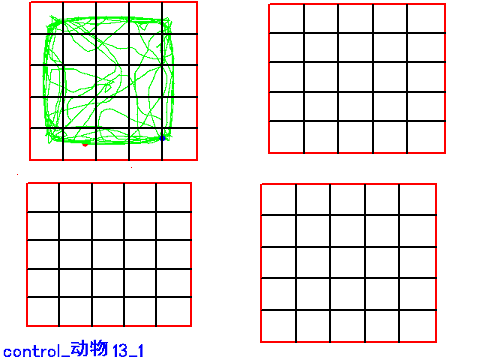


**LPS-1 d 5**


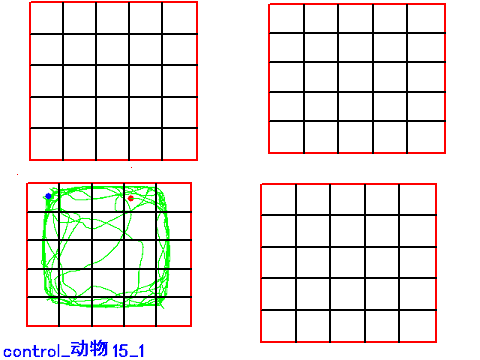


**LPS-1 d 6**


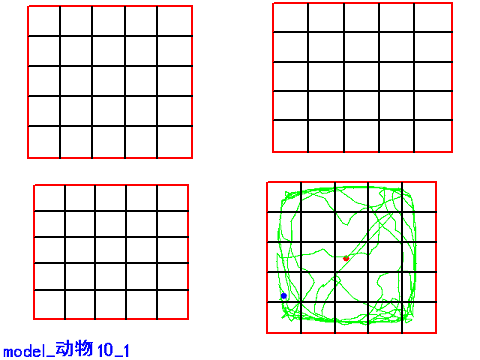


**LPS-1 d 7**


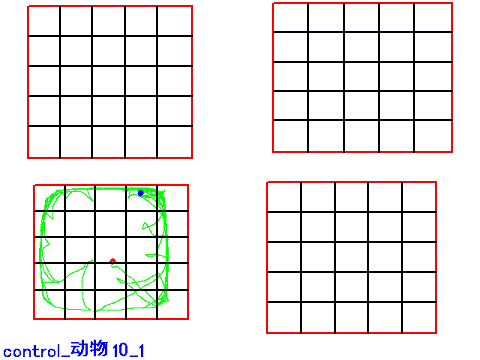


**LPS-1 d 8**


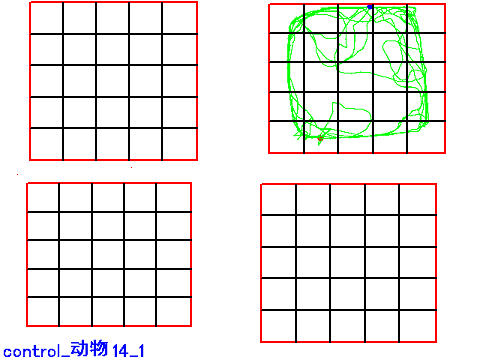


**LPS-1 d 9**


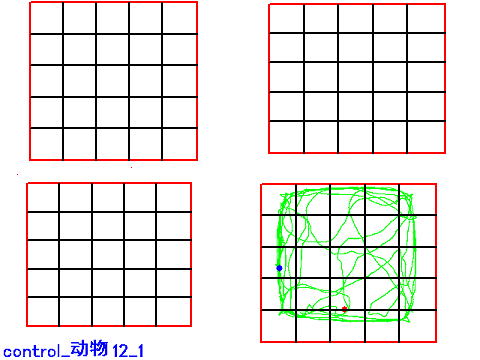


**LPS-1 d 10**


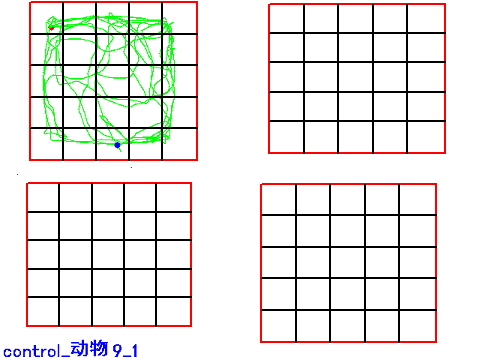


**LPS-2 d 1**


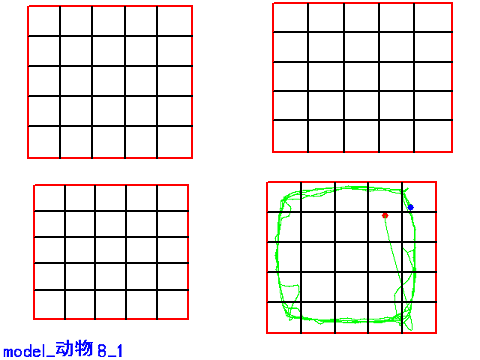


**LPS-2 d 2**


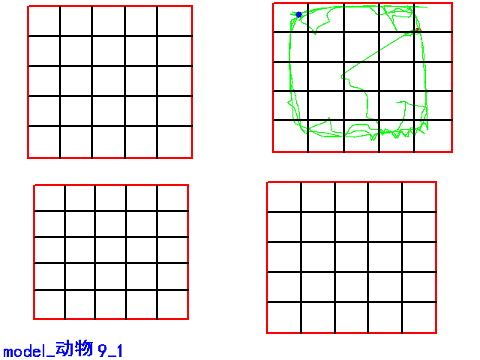


**LPS-2 d 3**


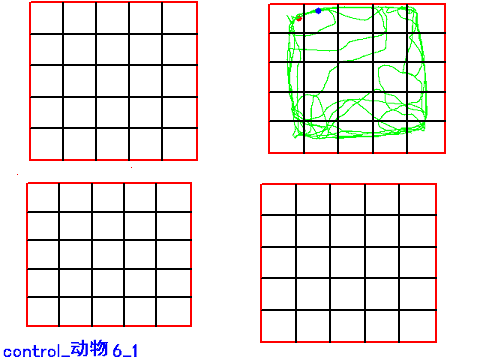


**LPS-2 d 4**


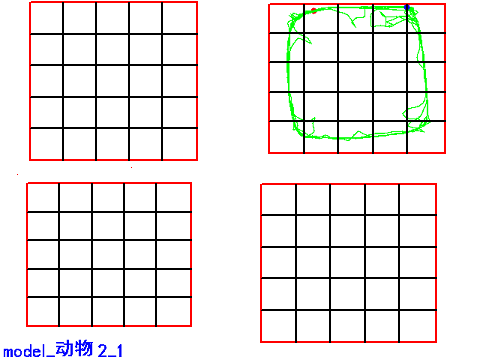


**LPS-2 d 5**


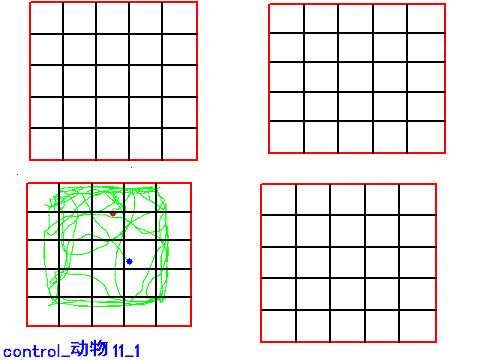


**LPS-2 d 6**


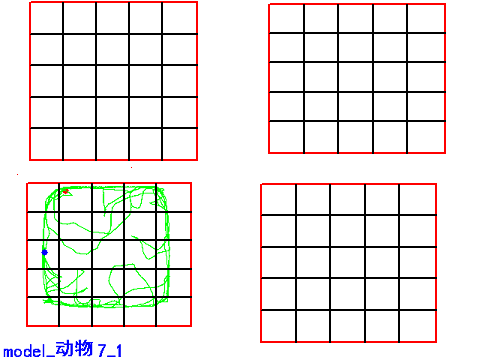


**LPS-2 d 7**


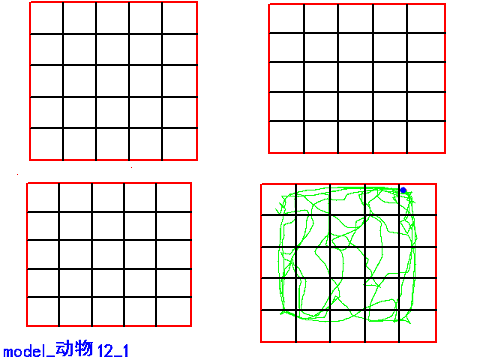


**LPS-2 d 8**


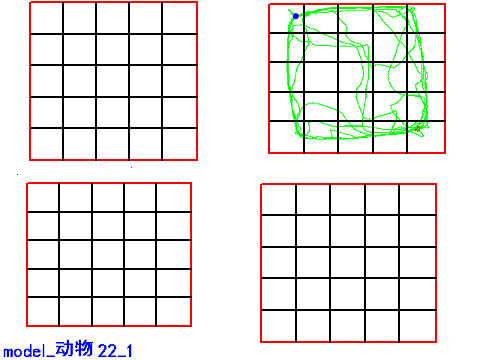


**LPS-2 d 9**


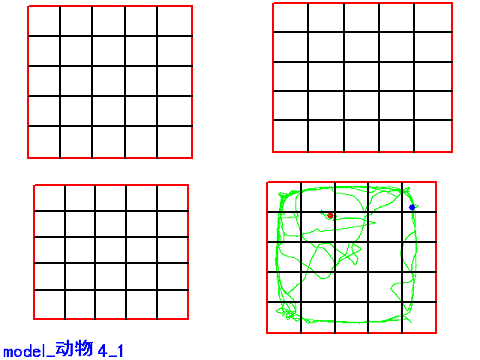


**LPS-2 d 10**


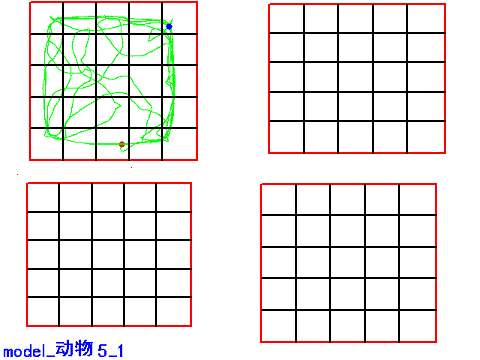


**LPS-4 d 1**


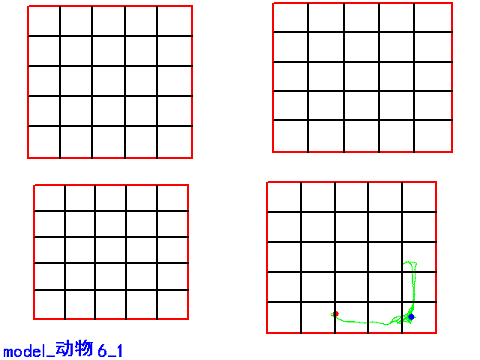


**LPS-4 d 2**


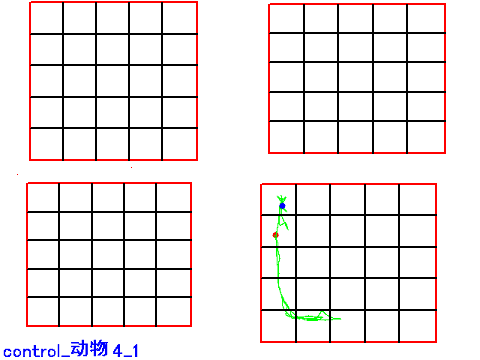


**LPS-4 d 3**


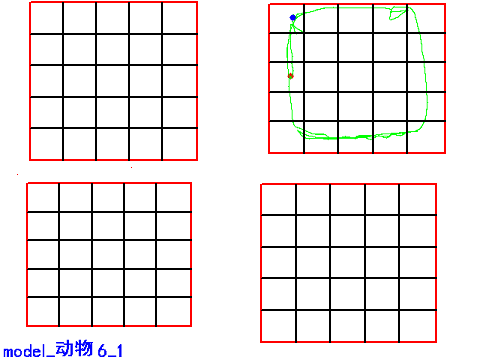


**LPS-4 d 4**


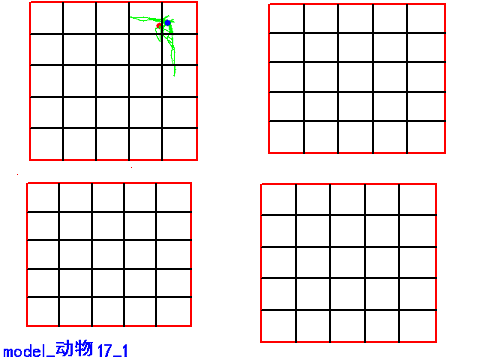


**LPS-4 d 5**


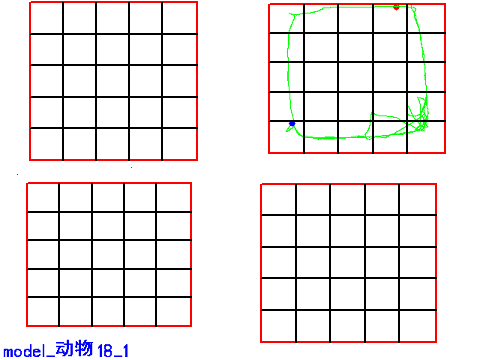


**LPS-4 d 6**


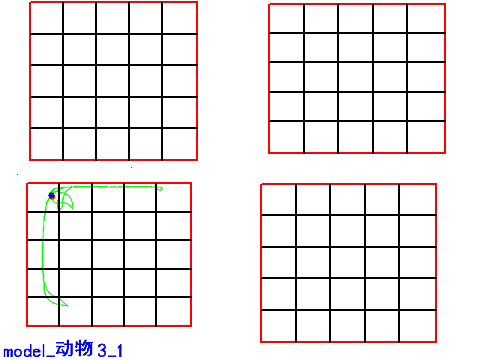


**LPS-4 d 7**


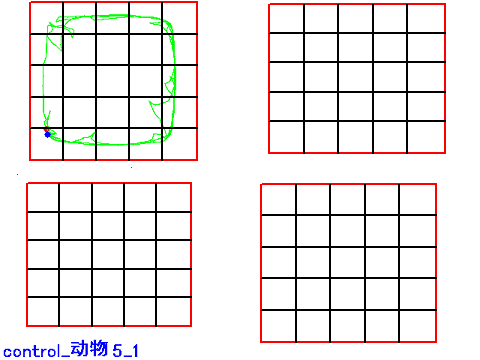


**LPS-4 d 8**


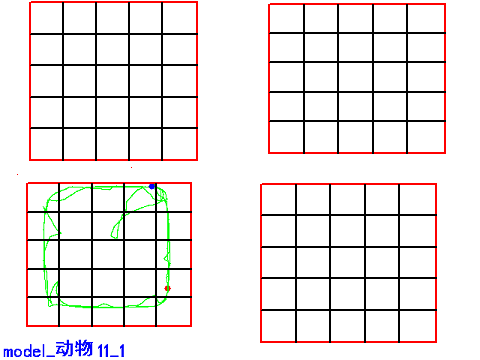


**LPS-4 d 9**


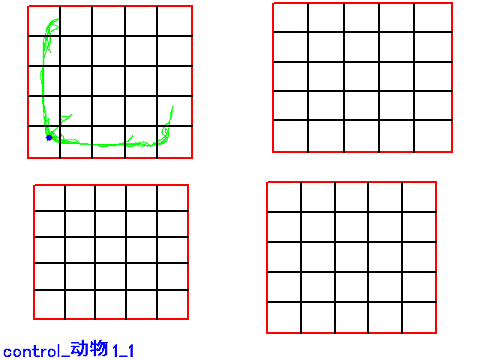


**LPS-4 d 10**


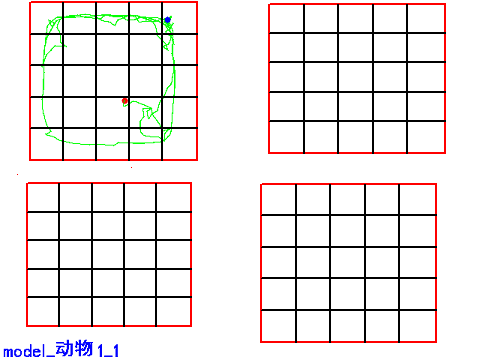

Supplement: Supplementary file 1 [file DataSheet1.ZIP › Behavioral tests/Trajectory Chart.docx]
