# Supplementary figures and images for: LPS activates neuroinflammatory pathways to induce depression in Parkinson’s disease-like condition
Source: Front Pharmacol. 2022 Oct 6;13:961817. doi: 10.3389/fphar.2022.961817 (PMC9582846; doi:10.3389/fphar.2022.961817)

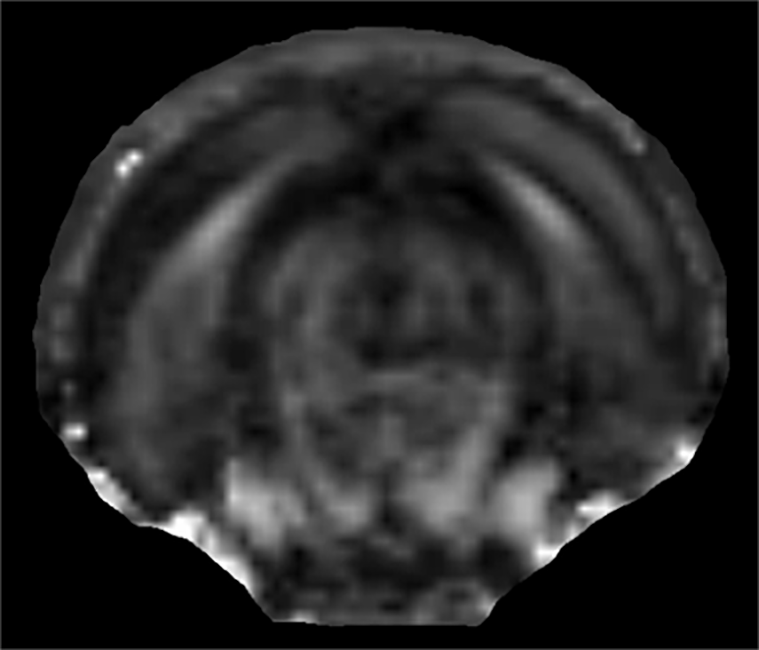

Supplement: Supplementary file 1 [file DataSheet1.ZIP › DTI results/FA/control 1-DRN.tif]

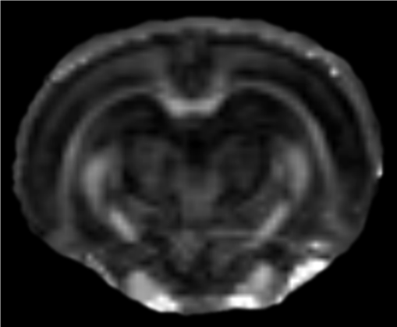

Supplement: Supplementary file 1 [file DataSheet1.ZIP › DTI results/FA/control 1-PFC,HIP,VTA.tif]

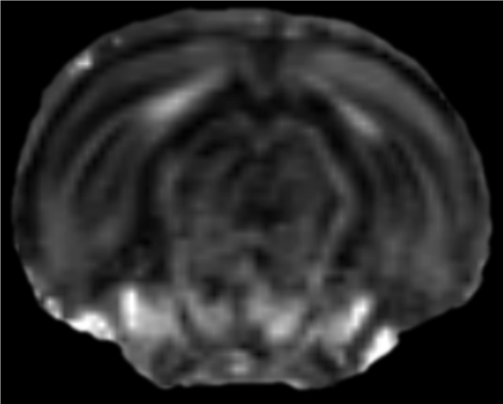

Supplement: Supplementary file 1 [file DataSheet1.ZIP › DTI results/FA/control 2-DRN.tif]

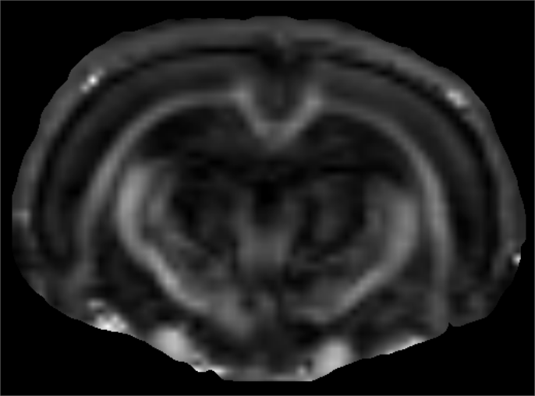

Supplement: Supplementary file 1 [file DataSheet1.ZIP › DTI results/FA/control 2-PFC,HIP,VTA.tif]

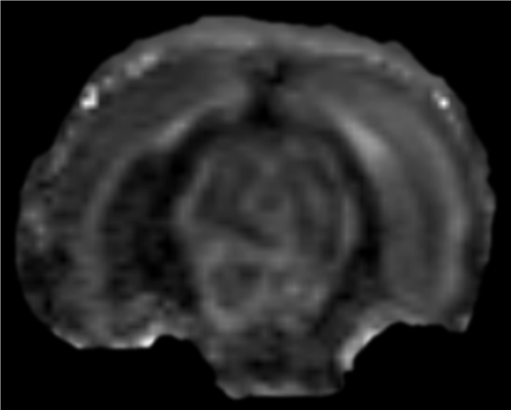

Supplement: Supplementary file 1 [file DataSheet1.ZIP › DTI results/FA/control 3-DRN.tif]

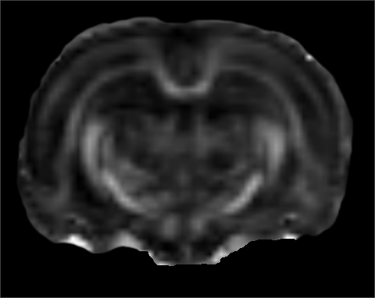

Supplement: Supplementary file 1 [file DataSheet1.ZIP › DTI results/FA/control 3-PFC,HIP,VTA.tif]

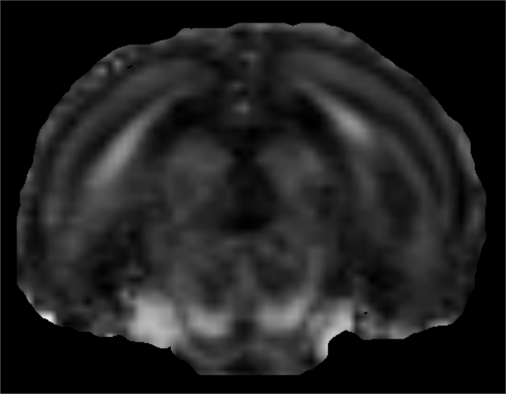

Supplement: Supplementary file 1 [file DataSheet1.ZIP › DTI results/FA/control 4-DRN.tif]

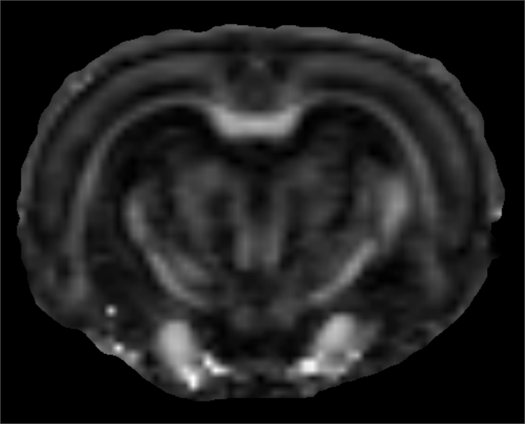

Supplement: Supplementary file 1 [file DataSheet1.ZIP › DTI results/FA/control 4-PFC,HIP,VTA.tif]

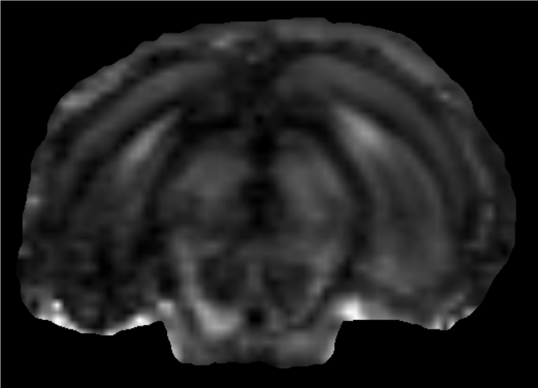

Supplement: Supplementary file 1 [file DataSheet1.ZIP › DTI results/FA/control 5-DRN.tif]

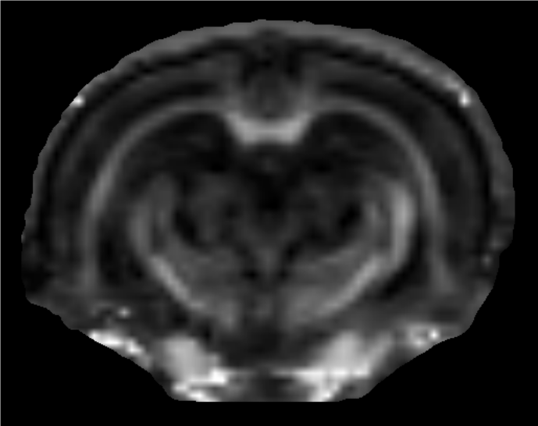

Supplement: Supplementary file 1 [file DataSheet1.ZIP › DTI results/FA/control 5-PFC,HIP,VTA.tif]

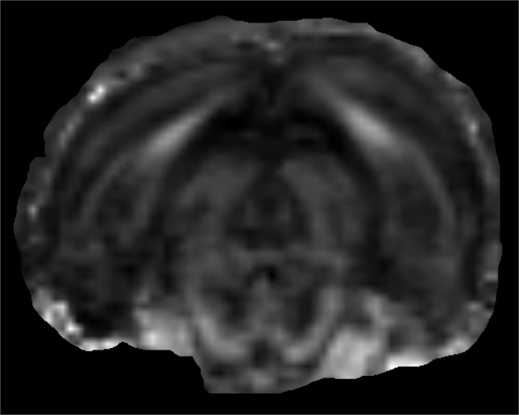

Supplement: Supplementary file 1 [file DataSheet1.ZIP › DTI results/FA/control 6-DRN.tif]

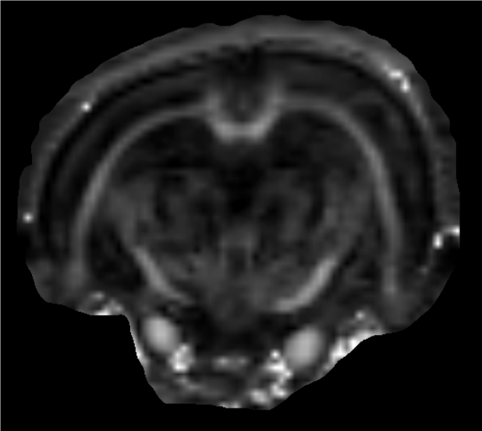

Supplement: Supplementary file 1 [file DataSheet1.ZIP › DTI results/FA/control 6-PFC,HIP,VTA.tif]

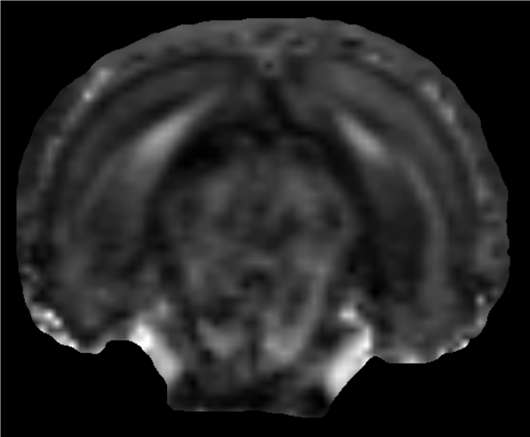

Supplement: Supplementary file 1 [file DataSheet1.ZIP › DTI results/FA/LPS-4 d 1-DRN.tif]

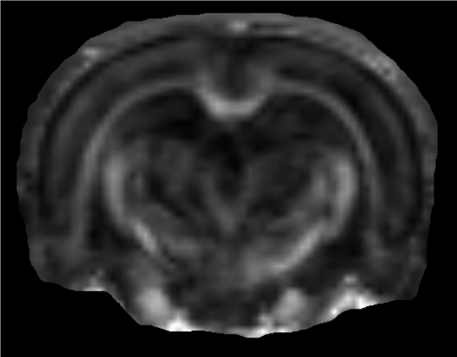

Supplement: Supplementary file 1 [file DataSheet1.ZIP › DTI results/FA/LPS-4 d 1-PFC,HIP,VTA.tif]

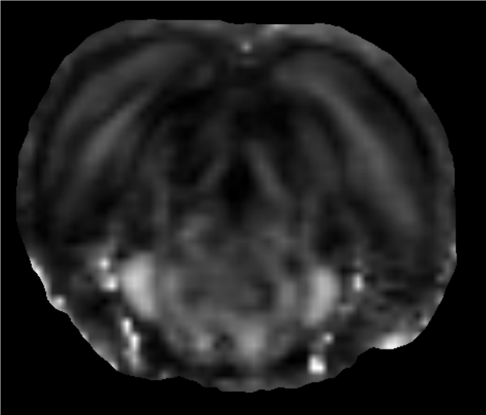

Supplement: Supplementary file 1 [file DataSheet1.ZIP › DTI results/FA/LPS-4 d 2-DRN.tif]

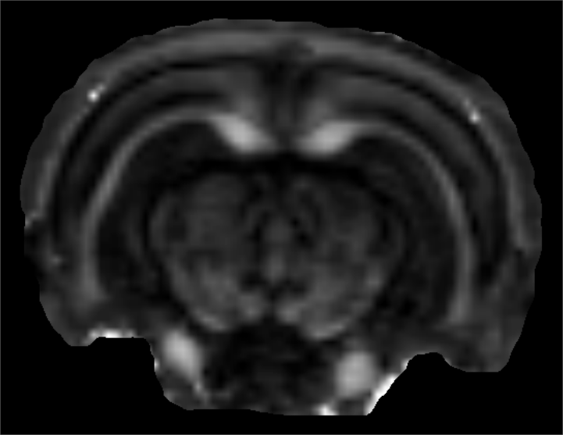

Supplement: Supplementary file 1 [file DataSheet1.ZIP › DTI results/FA/LPS-4 d 2-PFC,HIP,VTA.tif]

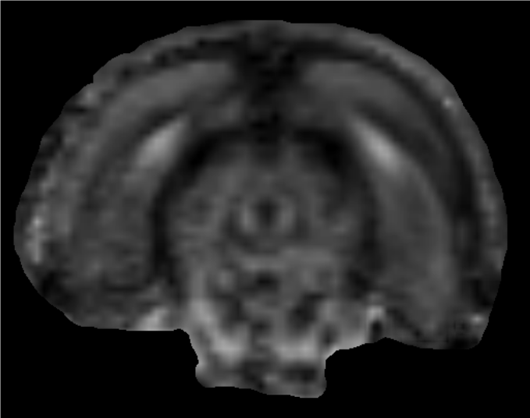

Supplement: Supplementary file 1 [file DataSheet1.ZIP › DTI results/FA/LPS-4 d 3-DRN.tif]

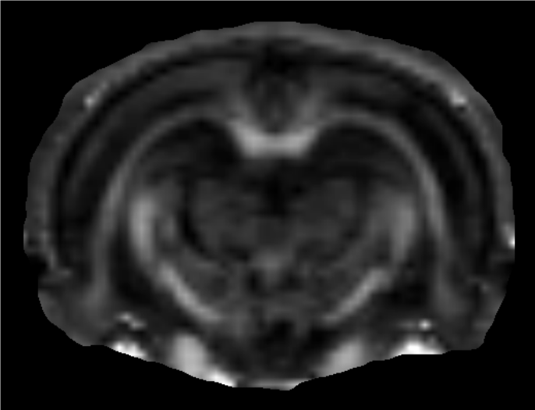

Supplement: Supplementary file 1 [file DataSheet1.ZIP › DTI results/FA/LPS-4 d 3-PFC,HIP,VTA.tif]

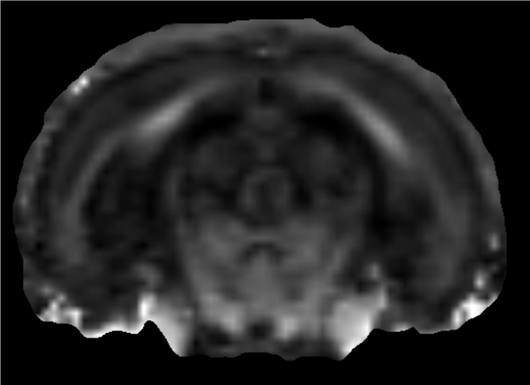

Supplement: Supplementary file 1 [file DataSheet1.ZIP › DTI results/FA/LPS-4 d 4-DRN.tif]

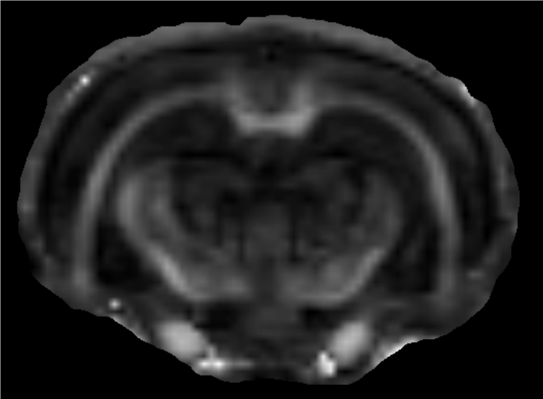

Supplement: Supplementary file 1 [file DataSheet1.ZIP › DTI results/FA/LPS-4 d 4-PFC,HIP,VTA.tif]

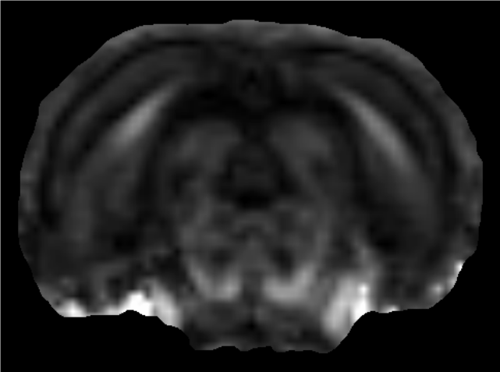

Supplement: Supplementary file 1 [file DataSheet1.ZIP › DTI results/FA/LPS-4 d 5-DRN.tif]

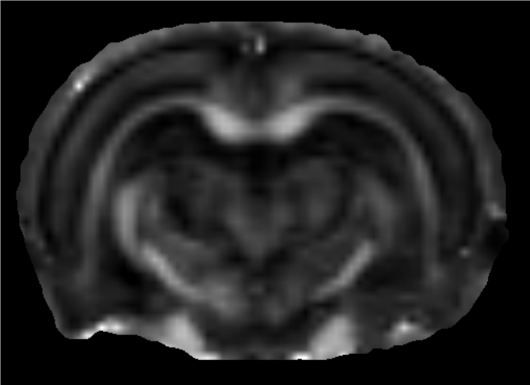

Supplement: Supplementary file 1 [file DataSheet1.ZIP › DTI results/FA/LPS-4 d 5-PFC,HIP,VTA.tif]

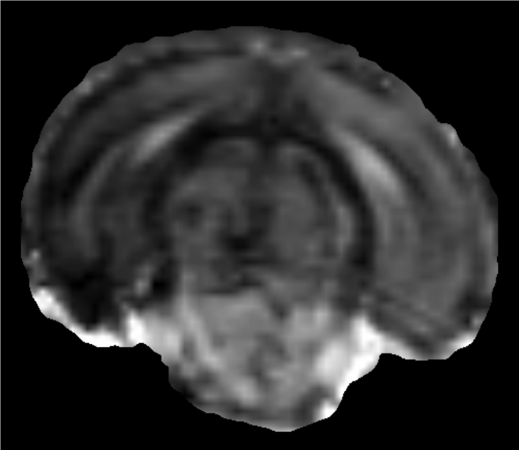

Supplement: Supplementary file 1 [file DataSheet1.ZIP › DTI results/FA/LPS-4 d 6-DRN.tif]

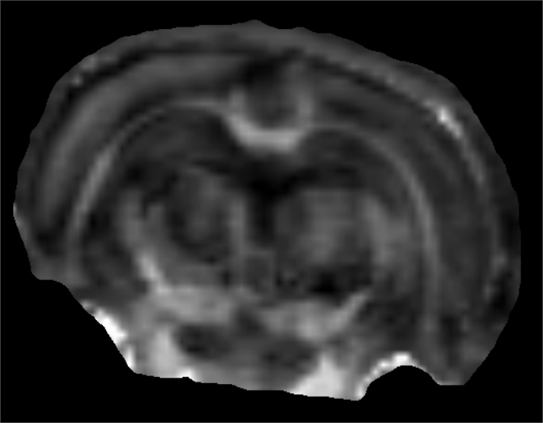

Supplement: Supplementary file 1 [file DataSheet1.ZIP › DTI results/FA/LPS-4 d 6-PFC,HIP,VTA.tif]

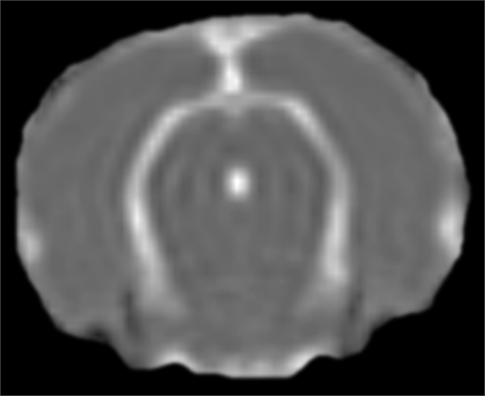

Supplement: Supplementary file 1 [file DataSheet1.ZIP › DTI results/MD/control 1-DRN.tif]

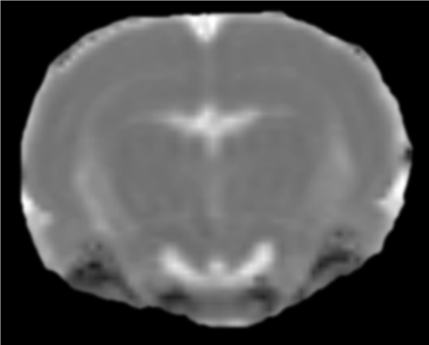

Supplement: Supplementary file 1 [file DataSheet1.ZIP › DTI results/MD/control 1-PFC,HIP,VTA.tif]

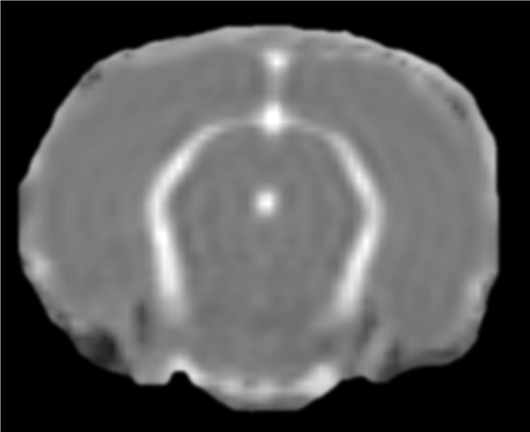

Supplement: Supplementary file 1 [file DataSheet1.ZIP › DTI results/MD/control 2-DRN.tif]

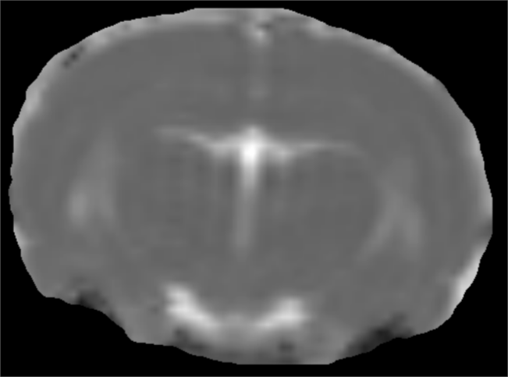

Supplement: Supplementary file 1 [file DataSheet1.ZIP › DTI results/MD/control 2-PFC,HIP,VTA.tif]

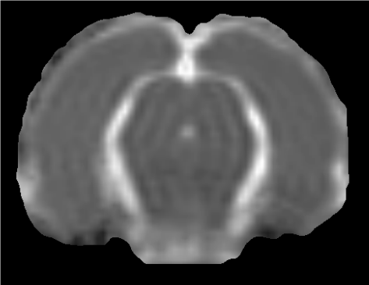

Supplement: Supplementary file 1 [file DataSheet1.ZIP › DTI results/MD/control 3-DRN.tif]

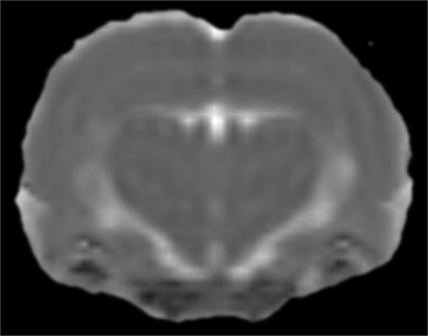

Supplement: Supplementary file 1 [file DataSheet1.ZIP › DTI results/MD/control 3-PFC,HIP,VTA.tif]

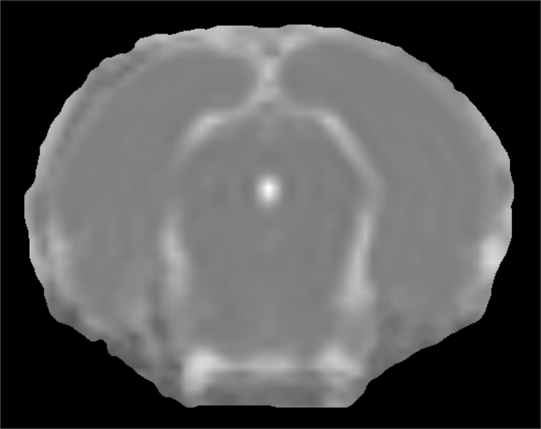

Supplement: Supplementary file 1 [file DataSheet1.ZIP › DTI results/MD/control 4-DRN.tif]

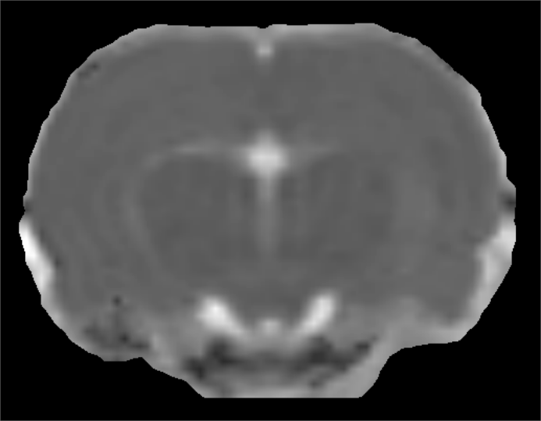

Supplement: Supplementary file 1 [file DataSheet1.ZIP › DTI results/MD/control 4-PFC,HIP,VTA.tif]

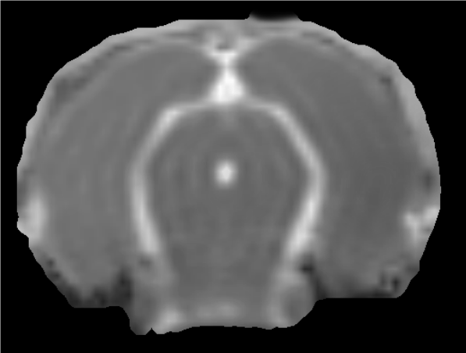

Supplement: Supplementary file 1 [file DataSheet1.ZIP › DTI results/MD/control 5-DRN.tif]

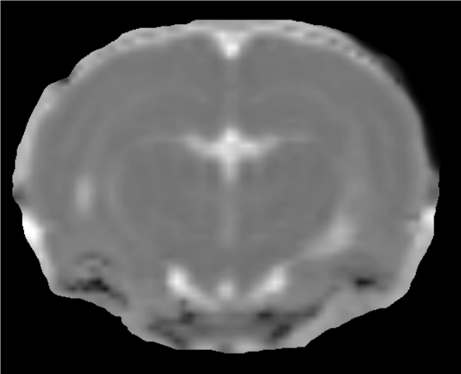

Supplement: Supplementary file 1 [file DataSheet1.ZIP › DTI results/MD/control 5-PFC,HIP,VTA.tif]

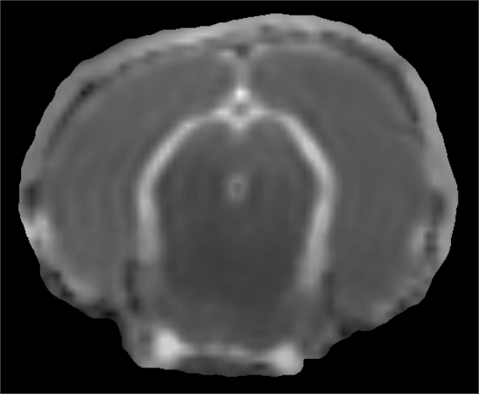

Supplement: Supplementary file 1 [file DataSheet1.ZIP › DTI results/MD/control 6-DRN.tif]

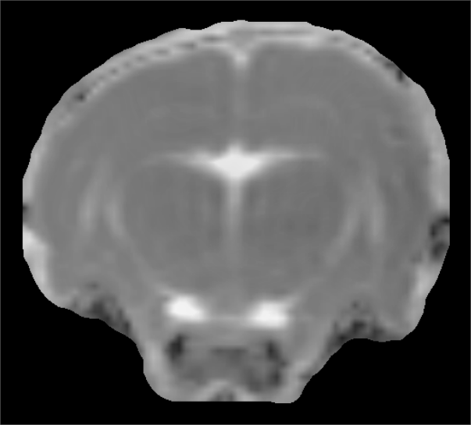

Supplement: Supplementary file 1 [file DataSheet1.ZIP › DTI results/MD/control 6-PFC,HIP,VTA.tif]

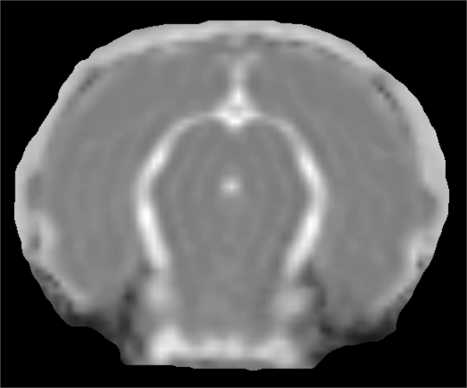

Supplement: Supplementary file 1 [file DataSheet1.ZIP › DTI results/MD/LPS-4 d 1-DRN.tif]

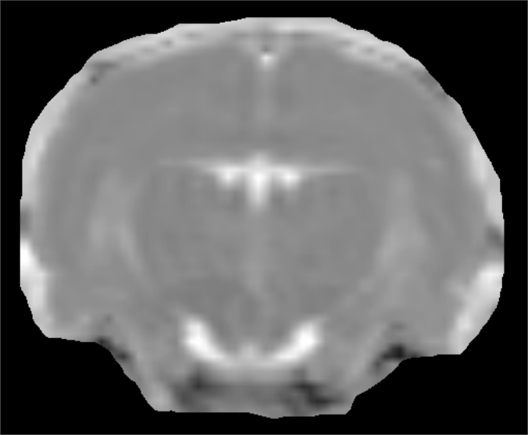

Supplement: Supplementary file 1 [file DataSheet1.ZIP › DTI results/MD/LPS-4 d 1-PFC,HIP,VTA.tif]

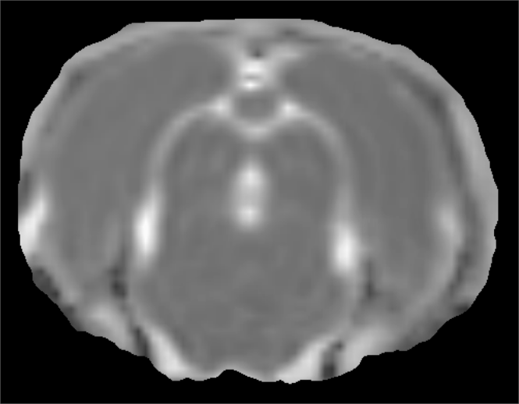

Supplement: Supplementary file 1 [file DataSheet1.ZIP › DTI results/MD/LPS-4 d 2-DRN.tif]

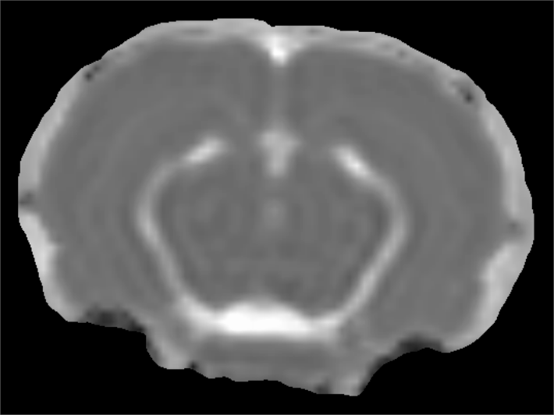

Supplement: Supplementary file 1 [file DataSheet1.ZIP › DTI results/MD/LPS-4 d 2-PFC,HIP,VTA.tif]

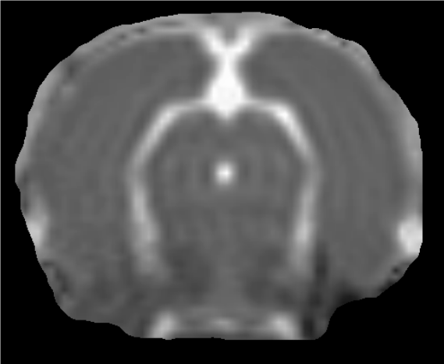

Supplement: Supplementary file 1 [file DataSheet1.ZIP › DTI results/MD/LPS-4 d 3-DRN.tif]

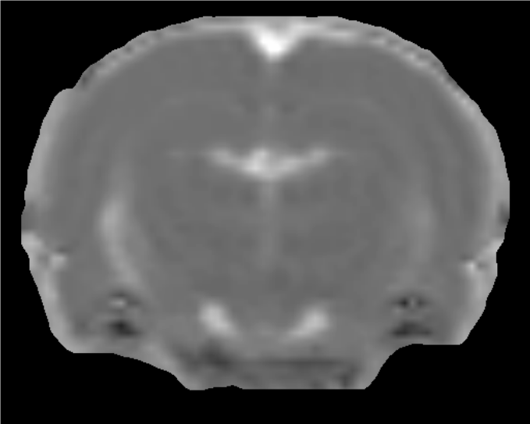

Supplement: Supplementary file 1 [file DataSheet1.ZIP › DTI results/MD/LPS-4 d 3-PFC,HIP,VTA.tif]

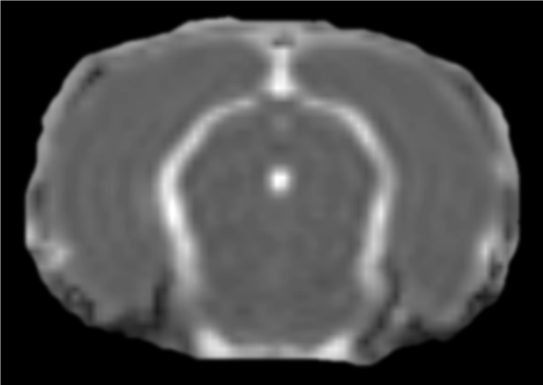

Supplement: Supplementary file 1 [file DataSheet1.ZIP › DTI results/MD/LPS-4 d 4-DRN.tif]

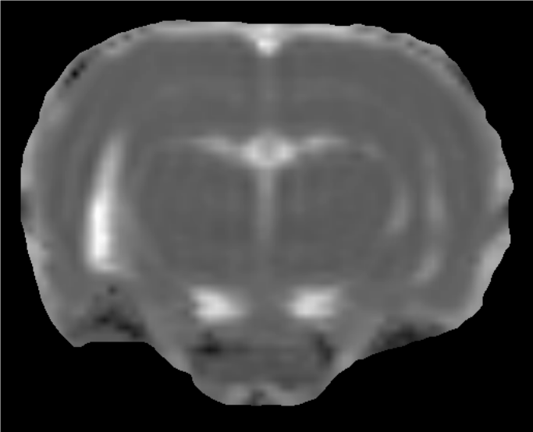

Supplement: Supplementary file 1 [file DataSheet1.ZIP › DTI results/MD/LPS-4 d 4-PFC,HIP,VTA.tif]

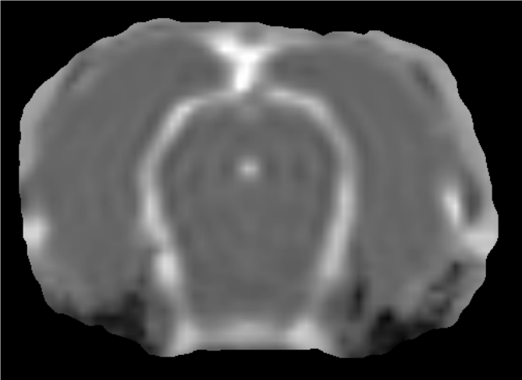

Supplement: Supplementary file 1 [file DataSheet1.ZIP › DTI results/MD/LPS-4 d 5-DRN.tif]

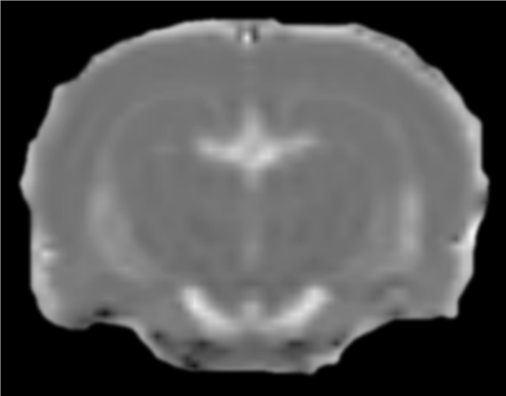

Supplement: Supplementary file 1 [file DataSheet1.ZIP › DTI results/MD/LPS-4 d 5-PFC,HIP,VTA.tif]

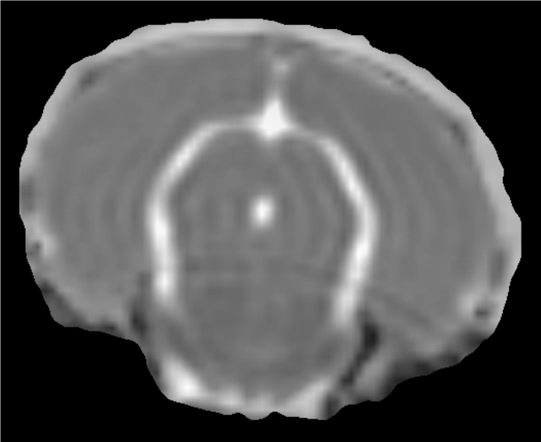

Supplement: Supplementary file 1 [file DataSheet1.ZIP › DTI results/MD/LPS-4 d 6-DRN.tif]

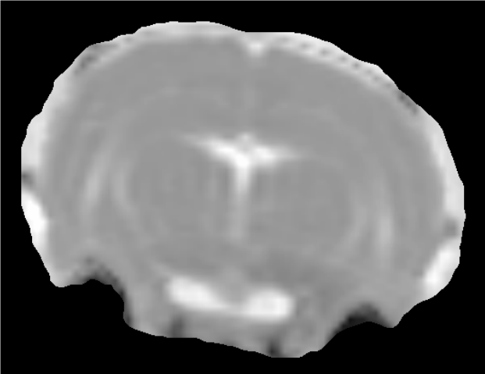

Supplement: Supplementary file 1 [file DataSheet1.ZIP › DTI results/MD/LPS-4 d 6-PFC,HIP,VTA.tif]

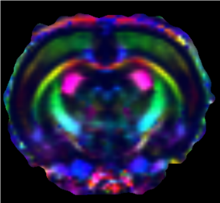

Supplement: Supplementary file 1 [file DataSheet1.ZIP › DTI results/Tracing of nerve fibers/control 1.tif]

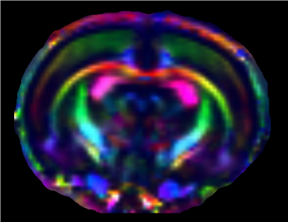

Supplement: Supplementary file 1 [file DataSheet1.ZIP › DTI results/Tracing of nerve fibers/control 2.tif]

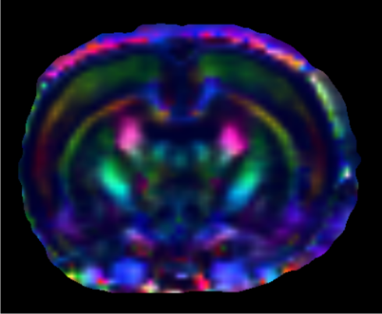

Supplement: Supplementary file 1 [file DataSheet1.ZIP › DTI results/Tracing of nerve fibers/control 3.tif]

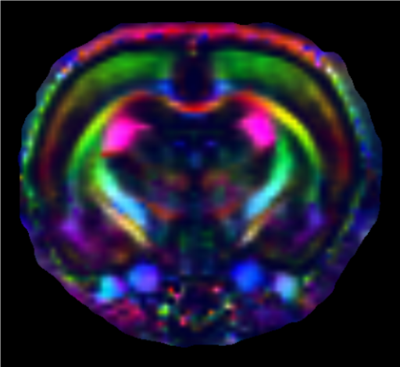

Supplement: Supplementary file 1 [file DataSheet1.ZIP › DTI results/Tracing of nerve fibers/control 4.tif]

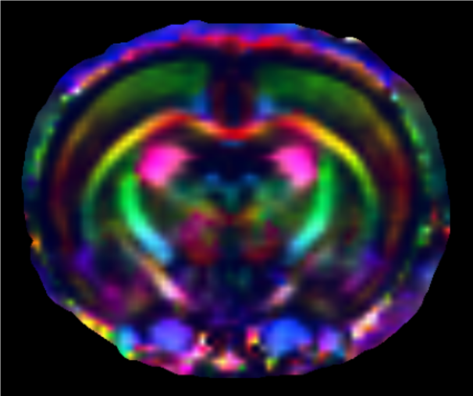

Supplement: Supplementary file 1 [file DataSheet1.ZIP › DTI results/Tracing of nerve fibers/control 5.tif]

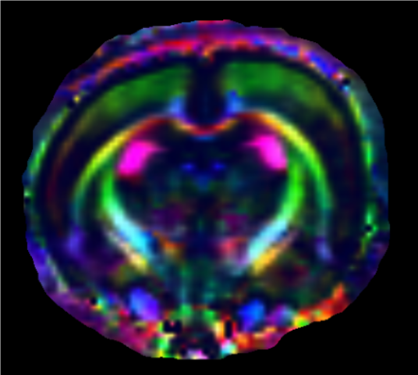

Supplement: Supplementary file 1 [file DataSheet1.ZIP › DTI results/Tracing of nerve fibers/control 6.tif]

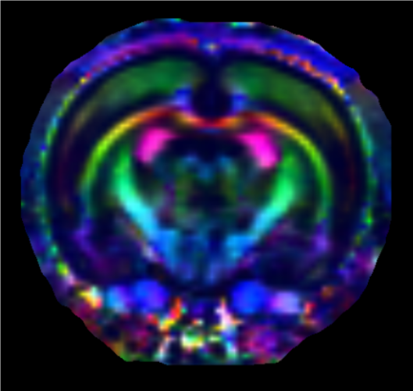

Supplement: Supplementary file 1 [file DataSheet1.ZIP › DTI results/Tracing of nerve fibers/LPS-4 d 1.tif]

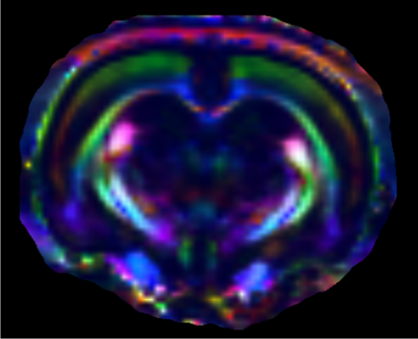

Supplement: Supplementary file 1 [file DataSheet1.ZIP › DTI results/Tracing of nerve fibers/LPS-4 d 2.tif]

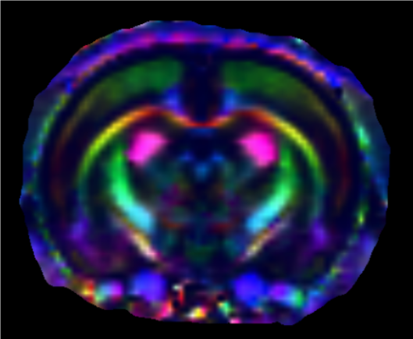

Supplement: Supplementary file 1 [file DataSheet1.ZIP › DTI results/Tracing of nerve fibers/LPS-4 d 3.tif]

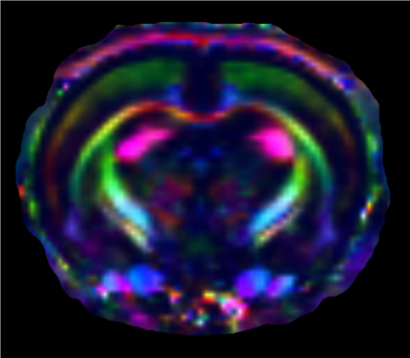

Supplement: Supplementary file 1 [file DataSheet1.ZIP › DTI results/Tracing of nerve fibers/LPS-4 d 4.tif]

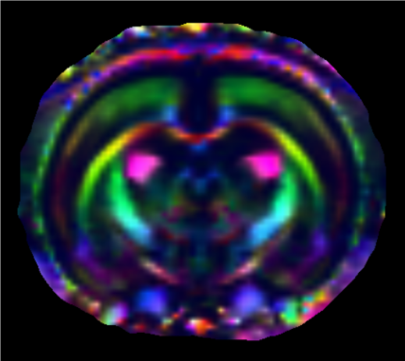

Supplement: Supplementary file 1 [file DataSheet1.ZIP › DTI results/Tracing of nerve fibers/LPS-4 d 5.tif]

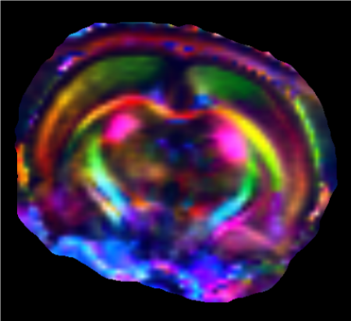

Supplement: Supplementary file 1 [file DataSheet1.ZIP › DTI results/Tracing of nerve fibers/LPS-4 d 6.tif]

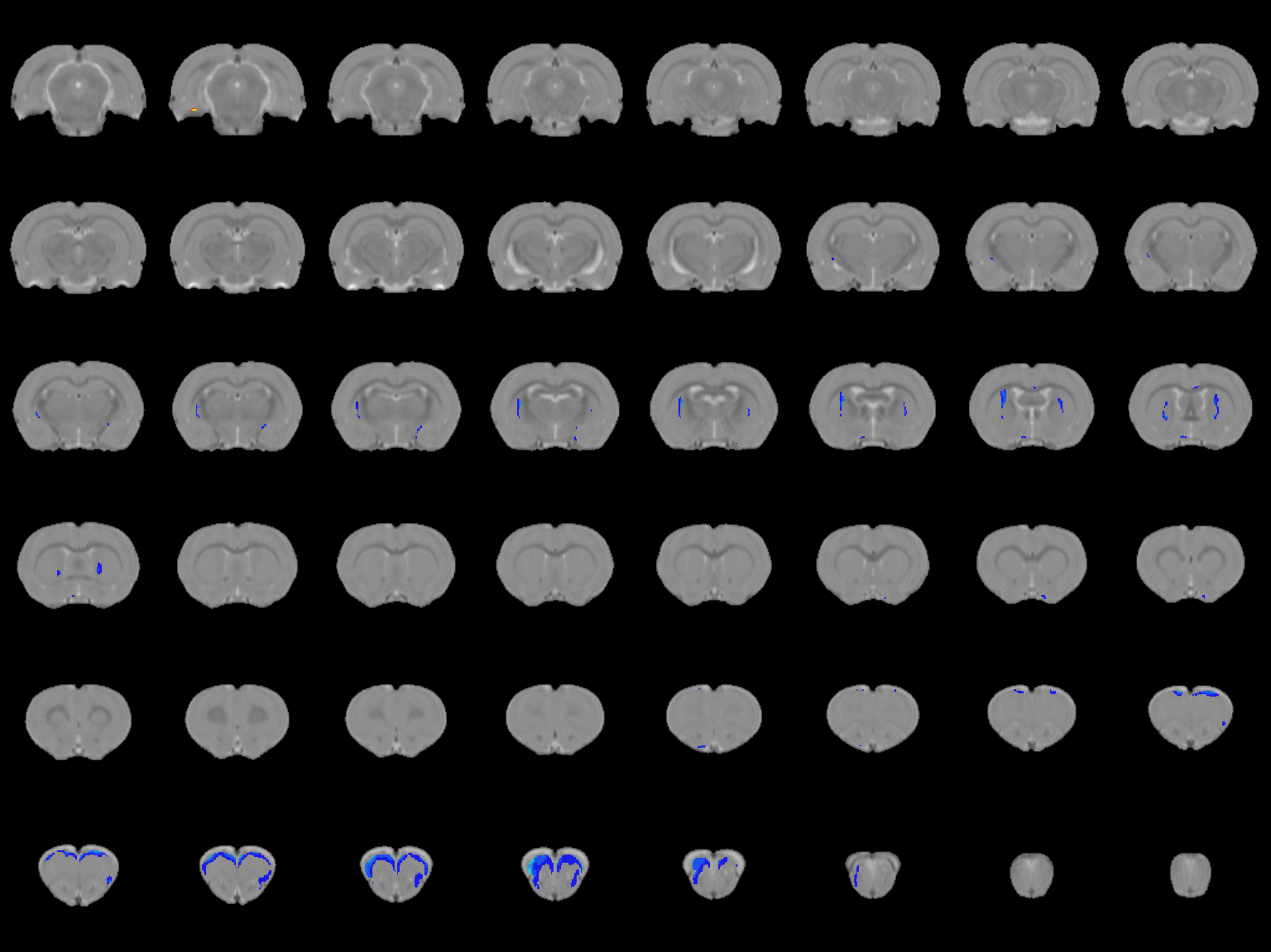

Supplement: Supplementary file 1 [file DataSheet1.ZIP › Supplementary Figure 1 A voxel based morphometry study between the control and LPS-4 d groups.tif]
